# Genomic Porosity between Invasive *Chondrostoma nasus* and Endangered Endemic *Parachondrostoma toxostoma* (Cyprinidae): The Evolution of MHC IIB Genes

**DOI:** 10.1371/journal.pone.0065883

**Published:** 2013-06-18

**Authors:** Andrea Šimková, Kristína Civáňová, Lenka Gettová, André Gilles

**Affiliations:** 1 Department of Botany and Zoology, Faculty of Science, Masaryk University, Brno, Czech Republic; 2 Aix-Marseille Université, Institut Méditerranéen de Biodiversité et d'Ecologie Marine et Continentale, UMR Centre national de la recherche scientifique 7263, Evolution Génome Environnement, Marseille, France; BiK-F Biodiversity and Climate Research Center, Germany

## Abstract

Two cyprinid species, *Parachondrostoma toxostom*a, an endemic threatened species, and *Chondrostoma nasus*, an invasive species, live in sympatry in southern France and form two sympatric zones where the presence of intergeneric hybrids is reported. To estimate the potential threat to endemic species linked to the introduction of invasive species, we focused on the DAB genes (functional MHC IIB genes) because of their adaptive significance and role in parasite resistance. More specifically, we investigated (1) the variability of MHC IIB genes, (2) the selection pattern shaping MHC polymorphism, and (3) the extent to which trans-species evolution and intergeneric hybridization affect MHC polymorphism.

In sympatric areas, the native species has more diversified MHC IIB genes when compared to the invasive species, probably resulting from the different origins and dispersal of both species. A similar level of MHC polymorphism was found at population level in both species, suggesting similar mechanisms generating MHC diversity. In contrast, a higher number of *DAB*-like alleles per specimen were found in invasive species. Invasive species tended to express the alleles of two *DAB* lineages, whilst native species tended to express the alleles of only the *DAB3* lineage. Hybrids have a pattern of MHC expression intermediate between both species. Whilst positive selection acting on peptide binding sites (PBS) was demonstrated in both species, a slightly higher number of positively selected sites were identified in *C. nasus*, which could result from parasite-mediated selection. Bayesian clustering analysis revealed a similar pattern of structuring for the genetic variation when using microsatellites or the MHC approach. We confirmed the importance of trans-species evolution for MHC polymorphism. In addition, we demonstrated bidirectional gene flow for MHC IIB genes in sympatric areas. The positive significant correlation between MHC and microsatellites suggests that demographic factors may contribute to MHC variation on a short time scale.

## Introduction

The focus on genetic variation at the major histocompatibility complex (MHC) represents a new perspective in recent conservation studies to estimate the threat to native species due to habitat modification or destruction, the introduction of non-native (invasive) species, and climate change. The functional MHC genes playing a critical role in immune response are especially suitable for genetic studies in the area of conservation focusing on endangered species (e.g. [1]–[4]) because of their adaptive significance and their role in pathogen or parasite resistance. Using a combination of specific functional MHC loci and genome-wide random markers is considered to be a good way to assess the potential threat to endemic species posed by reduced genetic diversity, because such an approach clarifies the patterns of adaptive variation and the role of selection in MHC evolution, and also gives information about demographic processes and the genetic structure of populations (e.g. [1], [5]–[8]).

As regards MHC, the molecules of this complex present pathogen- or parasite-derived peptides to T-cells that subsequently initiate a specific immune response. A typical feature of MHC genes is their extremely high level of polymorphism i.e. a high number of alleles coding the proteins and a high degree of nucleotide difference between alleles [9]. The high MHC polymorphism is most pronounced in the peptide-binding region (PBR), which is at specific amino-acid sites directly in contact with the bound peptides that mediate the recognition of the foreign antigens derived from pathogens and parasites. This polymorphism is maintained by several evolutionary mechanisms, such as balancing selection associated with the pressure exerted by pathogens and parasites (e.g. [10]–[11]); reproductive mechanisms such as, for instance, mating choice (e.g. [12]–[14]), gene duplication (e.g. [15]), and inter- and intra-locus recombination [16]; and spatially heterogeneous selective pressures [17]–[19]. In addition, MHC variability can also be affected by genetic drift on a short timescale [20].

Concerning balancing selection, two mechanisms maintain the high diversity of MHC genes at population level. The first one is overdominant selection conferring resistance on heterozygotes [21]. The heterozygotes at MHC genes are able to recognize a wider range of antigens derived from pathogens or parasites than the homozygotes (this was confirmed for fish e.g. by Hedrick *et al*. [1]). The second mechanism that maintains long-term polymorphism is the negative frequency-dependent selection giving an advantage to specimens with a rare allele [10]. In this case, the initially rare MHC allele conferring resistance against a given pathogen is favoured by selection and this allele becomes established in the population. The frequencies of MHC alleles in the population constantly change with the frequency of adapted and non-adapted pathogens.

The idea of the trans-species evolution of MHC polymorphism is generally accepted, i.e. the passage of allelic lineages from ancestral to descendent species is the mechanism generating the sharing of MHC alleles between closely related species [22]. However, in the case of hybridizing species, MHC polymorphism could also result from genetic introgression, where MHC alleles arising in one species after speciation introgress into the other species by hybridization [23]. Nadachovska-Brzyska *et al*. [24] showed that interspecific hybridization between two sister species of newts (*Lissotriton vulgaris* and *L. montadoni*) increased MHC class II diversity, and hypothesized that the interspecific exchange of MHC genes may be facilitated by frequency-dependent selection. In contrast, they showed that geographically separated populations of *L. vulgaris* were well differentiated in MHC genes, indicating local adaptation and low gene flow. Wegner and Eizaguirre [23] hypothesized that both parasite-mediated and sexual selection may contribute to the introgression of MHC alleles into the genome of related species, and both mechanisms, i.e. negative frequency dependent selection and overdominant selection, can drive the evolution of MHC introgression.

Concerning cyprinid fish, hybridization and genetic introgression are relatively common phenomena [25]. Such hybridization has resulted from the past introduction of new species and has led to the endangerment of native species. We are faced with such a situation in southern France for two cyprinid species, the native *Parachondrostoma toxostoma* and the introduced *Chondrostoma nasus*. In two sympatric zones (the Durance and Ardeche Rivers, Rhone River drainage) the presence of hybrids was systematically observed and well documented [26]–[28]. *Parachondrostoma toxostoma* is a threatened and protected endemic species in southern France, whilst *Chondrostoma nasus* was introduced from Eastern Europe 150 years ago. The Durance hybrid zone has been more intensively studied than the Ardeche hybrid zone. The observed pattern in the Durance hybrid zone is around 60 years old and represents a complex system with multiple effects including inter-species competition, bidirectional introgression, and environmental pressures [28]. The structuring of the hybrid zone was linked to progressive urbanization along the Durance River with the construction of weirs and dams, which led to the alteration and destruction of natural habitats for fish species.

On the basis of mtDNA and four nuclear introns, Costedoat *et al*. [28] identified a wide range of *P. toxostoma* and *C. nasus* hybrids in the Durance hybrid zone. Costedoat *et al*. [27] demonstrated the mosaic character of this hybrid zone arising from the different proportions of two species along a spatial scale. Both species as well as their hybrids exhibit more diversified feeding behaviour in sympatric zones when compared to allopatric populations [29]. In allopatric zones, both species are morphologically and ecologically differentiated; *C. nasus* is a specialized perilithon feeder (i.e. feeding mainly on diatoms and algae), whilst *P. toxostoma* exhibits more generalist dietary behaviour including also the consumption of invertebrates [30]. Interestingly, the hybrids exhibit “super *P. toxostoma*” feeding behaviour, i.e. they feed on fewer diatoms and more invertebrates than both pure species [31]. Šimková *et al*. [32] showed that invasive *C. nasus* is more parasitized by ectoparasites (especially by monogeneans of *Dactylogyrus* species) than native *P. toxostoma*. They hypothesized that the intensity of infection is likely the result of co-evolutionary interactions between *C. nasus* and *Dactylogyrus* species. In addition, they suggested that *C. nasus* is a source of monogenean parasite infection for *P. toxostoma* in areas where both species live in sympatry, but that *P. toxostoma* is only weakly susceptible to *Dactylogyrus* infection.

The aims of this study were (1) to analyze the variability of MHC IIB genes in allopatric populations and sympatric zones of *P. toxostoma* and *C. nasus* in order to compare genetic differentiation within species (allopatric and sympatric populations) and between species, (2) to analyze the pattern of selection at MHC IIB genes in both species, hypothesizing that both selection and drift affect MHC variation, and (3) to investigate how trans-species evolution and/or intergeneric hybridization affects MHC variability. This study is a necessary precursor to analyses of associations between parasitism and the MHC IIB allelic repertory in two sympatric living cyprinid species.

## Materials and Methods

### Fish collection

A total of 233 specimens of *Parachondrostoma toxostoma*, *Chondrostoma nasus* and their respective hybrids were collected in Southern France in August 2008, June 2010, September 2010, and June 2011. One allopatric population of *P. toxostoma* from the Orbieu River (Mediterranean Coastal River) and one allopatric population of *C. nasus* from the Allier River (Loire drainage) were sampled. In addition, four localities situated in the Durance hybrid zone (Avignon, Manosque, and Pertuis on the Durance River, and Pont de Laragne on the Buech River, a tributary of the Durance River) and two localities situated in the Ardeche hybrid zone (Saint Just and Labeaume) were sampled. The Directions Départementales des Territoires (DDT) from Alpes-de-Haute-Provence, Hautes-Alpes, Vaucluse, (N° 2010-961 and N° 2011-133), Ardèche (N°2010-253-8), Puy-de-Domes (N°2008-01-04) and Aude (N° 2003-3555 and ONEMA11-2008) districts issued the permits for fish catching in France, where fishing was done in collaboration with Officers of the French ONEMA (Office National de l'Eau et des Milieux Aquatiques). A fin sample of each fish specimen was preserved in 96% ethanol. The spleen of each individual was removed, transferred into 1.5 ml tubes with RNA*later™* Storage Solution (Sigma-Aldrich) and stored at −80°C. This study was approved by the Animal Care and Use Committee at the Faculty of Science, Masaryk University in Brno (Czech Republic).

### Molecular identification

All individuals were identified using the mitochondrial 5′ part of the cytochrome *b* gene according to Costedoat *et al*. [27] and nuclear markers (41 microsatellites distributed in five multiplex PCR kits, [33]) applying the Bayesian clustering method in NewHybrid 1.1 [34], see [32]. For microsatellites, amplification was performed as described in Šimková *et al*. [32]. Allele sizes were scored against an internal GeneScan-500 LIZ standard and genotypes were obtained using GeneMapper® 3.7 [35].

### MHC analyses

RNA extraction and reverse-transcription were performed as described in Ottová *et al*. [36]. The complete exon 2 of *DAB* genes (functional genes of MHC class II*B* in fish) was analyzed in this study. Exon 2 represents the most variable part of MHC IIB genes including the peptide binding regions (PBR). The primers specific for *DAB1*-like genes and those specific for *DAB3*-like genes of *P. toxostoma* and *C. nasus* were developed in this study. The exon 2 of *DAB1*-like genes was amplified using forward primer DAB1_EX2_3F (CTGCTTTCACTGGAGCAGCTA) annealing to position 48 (or alternatively 30) and reverse primer DAB1_EX2_2R (GTCTGCCACCAGCCTGAG) annealing to position 372 (or alternatively 354) of the reference sequence for the *DAB1* (or alternatively *DAB2*) gene in *Cyprinus carpio* (accession numbers Z49064 and Z49065). The exon 2 of *DAB3*-like genes was amplified using forward primer DAB3_EX2_1F (GCTTTCACTGGAACAGCTG) annealing to position 33 (or alternatively 40) and reverse primer DAB3_EX2_1R (ACAGCTGGATGATTGCCTT) annealing to position 364 (or alternatively 371) of the reference sequence for the *DAB3* (or alternatively *DAB4*) gene in *Cyprinus carpio* (accession numbers X95431 and X95434). These pairs of primers amplify the complete exon 2 (276 bp long) and partial exon 1 and exon 3 in both genes (Supporting Information S1).

PCR was performed in a 30 µl reaction volume using 30–50 ng of cDNA. The reaction mixture was comprised of a 1× *Taq*Buffer with (NH_4_)_2_SO_4_, 1.5 mM MgCl_2_, 0.2 mM of each dNTP, 0.3 µM (for *DAB1*) or 0.35 µM (for *DAB3*) of each primer of gene specific primer pair, and 1 U (for *DAB1*) or 0.75 U (for *DAB3*) of *Taq* DNA polymerase (Fermentas). The amplification of *DAB1* ran in 35 cycles under the following conditions: initial denaturation at 94°C/2 min; cycling at 94°C/30 s, 58°C/40 s, 72°C/1 min; and a final extension at 72°C/10 min. The amplification of *DAB3* ran in 30 cycles under the following conditions: initial denaturation at 95°C/4 min; cycling at 95°C/30 s, 56°C/30 s, 72°C/45 s; and a final extension at 72°C/10 min. PCR reactions were performed in a Mastercycler ep gradient thermocycler (Eppendorf). PCR products (342 bp and 350 bp for *DAB1*-like and *DAB3*-like genes, respectively) were visualized on 1% GoldView-stained agarose gel and purified using High Pure PCR Product Purification Kit (Roche). For the quality and heterozygosity controls, the purified PCR products were sequenced in both directions using the same primers as in the amplification reaction. Sequencing reactions were performed using the fluorescent chemistry of BigDye Terminator v3.1 Ready reaction Cycle Sequencing Kit (Applied Biosystems). The purified products of the sequencing reaction (BigDye X-Terminator Purification Kit; according to the manufacturer's protocol) were analyzed using an ABI 3130 Genetic Analyzer (Applied Biosystems by Life Technologies, Carlsbad, California) under the appropriate module. Sequences were analyzed using Sequencing Analysis version 5.2 (Applied Biosystems) and Sequencher version 5.0 (Gene Codes Corp., Ann Arbor, MI USA) software.

Successfully amplified samples were subsequently subjected to clonal 454 amplicon pyrosequencing to distinguish particular alleles (454 Titanium Roche technology; commercial customer service by Beckman Coulter Genomics SAS, France). For template preparation, the non-classical fusion primers consisted of a 454 Titanium amplicon adaptor, a 6 bp sequence tag, and a specific primer sequence, but only tag-modified genotyping primers for PCR amplification were designed; i.e. the tag consisting of 7 nucleotides was added at the 5′ end to each specific PCR primer. The precise conditions for designing the primers' tags were as follows: no homopolymer in the tag, at least 3 substitutions between any pair of tags, and no motif repetition inside the tag. The PERL script used to create and select the tags compatible with the mentioned rules is available on request. In total, 40 unique tags were defined (unpublished), out of which 12 tag-modified forward primers and 8 tag-modified reverse primers for each gene made up the unique primer combination for each sample in a 96-well plate. These 7 bp tags were used to assign 454 sequencing reads to individual samples in later analysis.

Samples were newly amplified, each individual sample employing a unique tagged primer combination. The PCR mixture composition, volume, and cycling conditions were the same as in standard PCR (described above). The PCR amplicons with tagged primers were 356 bp and 364 bp long (for *DAB1* and *DAB3*, respectively; including tagged primers). The quality of the amplified product was checked by electrophoresis on 1% GoldView-stained agarose gel. After purification (High Pure PCR Product Purification Kit, Roche), the products were quantified by a Nanodrop 8000 spectrophotometer (Thermo Scientific) and mixed together using equimolar quantities of PCR products according to the provider's requirements (minimum of 1 µg and 2E^+10^ molecules of purified material in the final pool). As the amplicons for both genes have similar lengths and sequences, all samples were pooled together in one single tube (up to 96 samples for each gene, i.e. a maximum of 192 amplicons for each run, including positive and non-template controls). The prepared amplicon pool was sent to the service provider Beckman Coulter Genomics, who performed quality control of the template, adaptors ligation, fragment library construction, run reading, and raw data delivery (Supporting Information S2).

To extract, visualize and analyze amplicon sequences, we used the user-friendly SESAME program web application package [37] for analyzing amplicon sequences obtained through NGS (next generation sequencing) technologies. The output of the sequence variants from SESAME was further analyzed in Sequencher 5.0 (Gene Codes Corporation) and compared with the Sanger electrophoretogram of the particular sample. On the basis of the unique tag combination for each sample, the corresponding reads were assigned to each particular sample, and, following the similarity of reads with the gene reference sequences, the reads for particular samples were assigned to two groups of *DAB*-like alleles (*DAB1*-like and *DAB3*-like) (with an eValue corresponding to 1E^−10^) using SESAME. In addition, SESAME assistant provided the alignment and trimming of sequences, the processing of uncorrected sequences, and the final correction of sequences (pre-set error rate 0.05). After analyzing each run (3 runs were analyzed in this study) the output including the number of analyzed samples, number of raw sequences, number of marker-assigned sequences, number of sample-assigned sequences, number of samples with sequences, and final number of assigned variants for the run was created. For a particular fish sample, a maximum number of 8 *DAB*-like alleles (because of the hypothetical duplication of the *DAB1*-like gene and *DAB3*-like gene) was expected. The number of sequences, number of sequence variants, frequency of a particular sequence variant for each particular fish sample, and frequency of a particular sequence variant in the whole run were obtained. Even though all amplicons were adjusted to the same concentrations before pooling, the reads for different samples varied from hundreds to thousands. The assigned sequence variants for a particular sample matched the Sanger sequencing output.

To distinguish true alleles from artefacts generated at different stages of MHC genotyping, we followed the procedures of Zagalska-Neubauer *et al*. [38] as applied by Nadachowska-Brzyska *et al*. [24]. Briefly, we excluded all variants containing indels causing frameshifts or present in one copy in all data sets. We considered all amplicons with sufficient coverage and we calculated the maximum per-amplicon frequency (MPAF) for each sequence variant. Each sequence variant with an MPAF of less than 5% was checked for the potential artefact (i.e. 1 bp substitution or recombination from other sequence variants in a given amplicon). The sequence variants present in ≥50% of the amplicon's reads were not explained by the artefacts and were considered as the true alleles in our study. A large proportion of true alleles were confirmed by two independent PCRs. Nevertheless, as Nadachowska-Brzyska *et al*. [24] confirmed, in a multiple populations approach, many true alleles are observed as singletons. Because using the method of 454 sequencing avoids the problem of recombination in contrast to Sanger cloning [39], the singletons may represent the true MHC alleles. In our study, all singletons were sequence variants with mean MPAF equal 24%. In addition, the genotyping of all specimens for which the singletons were found was repeated twice.

The specific alleles of *P. toxostoma* were termed as *Pato-DAB* alleles and the specific alleles of *C. nasus* were termed as *Chna-DAB* alleles following the nomenclature of Klein *et al*. [40]. If the two species shared the alleles, we applied the nomenclature used by Nadachowska-Brzyska *et al*. [24] for hybridizing species of newts i.e. the alleles shared in *P. toxostoma* and *C. nasus* and the alleles identified solely in their hybrids were termed as *Pctn-DAB* alleles. Concerning *DAB* genes in cyprinid fish, Dixon *et al*. [41] presumed that *DAB1* and *DAB3* sets of sequences represent two different loci which may or may not contain several genes (i.e. the terminology *DAB1*-like genes and *DAB3*-like genes following Rakus *et al*. [15] and Seifertová and Šimková [42] was therefore applied in our study). The new *DAB* alleles were deposited in EMBL under Accession numbers from HF969045 to HF969122.

### Phylogenetic analyses

Phylogenetic reconstructions were performed in PAUP* 4.0b10 for Microsoft Windows 95/NT [43] applying a distance method using a neighbour-joining (NJ) algorithm. A bootstrap analysis with 1000 replicates was performed to determine the reliability of the branching in the phylogenetic tree. The hierarchical likelihood ratio test (hLRT) implemented in ModelTest v. 3.6 [44] was used to determine the appropriate substitution model of sequence evolution that best fitted the dataset. The maximum likelihood (ML) analyses using a heuristic search with a tree bisection reconnection branch-swapping algorithm were performed separately for *P. toxostoma* and *C. nasus*. The resulting ML trees were used in the codon-based analyses of selection performed in PAML 4.3 [45] (see below).

### Recombination and selection analyses

GENECONV v.1.81a [46], which employs a substitution model to detect the most likely candidate fragments for recombination/gene conversion events between sequences, was applied. GENECONV defines a fragment as an aligned pair of segments between two sequences that is bounded by either two discordant sites, or else by one discordant site and an end of the alignment. GENECONV looks for inner fragments, which are evidence of a possible gene conversion event between the ancestors of two sequences in the alignment. GENECONV can also look for outer fragments, which are evidence of past gene conversion events that may have originated from outside of the alignment, or else from within the alignment but such that evidence of the source has been destroyed by later mutation or gene conversion. By default, GENECONV finds significant “inner” and “outer” sequence fragments. Global and pairwise statistical tests for recombination events were used with 10,000 permutations of the data to assess significance. In the case of multiple tests, the software has an integrated Bonferroni correction that minimizes the inflation of type I errors in many pairwise tests that are performed in a reasonably large sequence sample.

First, to detect the signatures of positive selection in *DAB*-like sequences, the relative rate of nonsynonymous (*d*_N_) and synonymous substitutions (*d*_S_) was calculated according to Nei and Gojobori [47], applying the correction of Jukes and Cantor [48] for multiple hits using MEGA 5 [49]. A significant excess of d_N_ substitution over d_S_ substitution is considered as convincing evidence of non-neutral evolution. A one-tailed Z test was performed for all codons, using only ABS (antigen-binding sites) codons or only non-ABS codons. The ABS codons were inferred from the structure of the human crystal model of the MHC class 2 *DRB1* locus [50].

Next, analyses to detect positive selection on a site-by-site basis were performed using a maximum likelihood approach in the CODEML program implemented in PAML 4.3 [45]. Different codon-based models incorporating selection (M3, M2a and M8) and not incorporating selection (M0, M1a and M7) were used to test for the presence of sites under selection, as recommended by Yang [45]. The models used the nonsynonymous/synonymous rate ratio (ω = *d*_N_/*d*_S_) as an indicator of selective pressure on the protein. According to Yang [45], all three pairs of models (i.e. M0 (one ratio) versus M3 (a discrete model involving three site classes for ω), M1a (nearly neutral) versus M2a (positive selection), and M7 (a β model which uses beta distribution) versus M8 (β and ω ratio estimated from the data)) appear to be particularly useful, forming three likelihood ratio tests of positive selection. A likelihood ratio test (LRT) was used to assess the significance of the differences between models. If the LRT statistic comparing two models indicates that the alternative models (M2a, M3 and M8) fit the data significantly better than the simpler models (M1a, M0 and M7), this suggests the presence of sites with ω>1 and the action of positive selection at specific sites in the protein-coding sequences [51]. Following the recommendation of Yang [45], the Bayes empirical Bayes (BEB) method was used to calculate the posterior probabilities for site classes and to identify sites under selection (the posterior means of ω for positively selected sites (PSS) are >1). BEB was implemented under models M2a and M8 only.

### Population genetic analyses

The population genetic structure based on MHC IIB genes was analyzed using the approach of Nadachowska-Brzyska *et al*. [24]. Binary coding was used to determine the presence/absence of a given *DAB*-like allele in specimens. Because we were not able to assign the alleles to loci (i.e. our results suggest the duplication of both *DAB1* and *DAB3* loci, see below), each allele was considered as a separate dominant locus in population genetic analyses. A Bayesian clustering method implemented in STRUCTURE 2.3.3 [52] was used to infer and delineate the most probable number of genetically homogenous groups of sampled specimens (i.e. K genetic clusters) with no a priori assumptions of population structure, and to assign specimens to the inferred groups based on allelic frequencies. Here, a method developed for dominant markers such as AFLP markers [53] was used. A model with correlated allele frequencies and allowing admixture was applied. Five independent runs for K ranging from 1 to 10 were performed, using 4×10^6^ Markov chain Monte Carlo replications with a burn-in period of 4×10^5^ chains. To infer the most appropriate number of genetic clusters in the data set, the ad hoc statistic *ΔK* method proposed by Evanno *et al*. [54] was used. The results of ten independent runs using a fixed number of most probable K were subsequently post-processed in CLUMPP 1.1.2 [55]. Pairwise F_st_ values between populations for MHC were estimated using Arlequin 3.5.1.3 [56] (1) by applying the method developed for dominant markers using binary encoded data and (2) from haplotype frequencies using the allele haplotype and number of individuals with a given allele for each population. 95% confidence intervals (CI) around F_st_ values were calculated by bootstrapping 20000 times over loci.

For microsatellites, the presence of null alleles was checked in MICRO-CHECKER [57]. FSTAT 2.9.3.2 [58] was used to test for deviations from the Hardy-Weinberg equilibrium (HWE). Significance levels of multiple testing were adjusted using a false discovery rate correction [59] in the QVALUE program [60]. Number of alleles (N_a_), allelic richness, and number of private alleles were computed in FSTAT 2.9.3.2 [58] and GenAlEx 6 [61] programs. Arlequin software was also used to calculate the pairwise F_st_ values between populations with N>8 and their 95% CI (Supporting Information S3 and S4). Population structure using microsatellite allele size data was further studied using a Bayesian clustering approach applying the same model as described above. ANCOVA was used to test the effect of species on the microsatellites (number of alleles and allelic richness) or on the MHC (number of alleles and mean number of alleles per specimen in population); sample size was included as covariate. The Spearman correlation was calculated between MHC allelic richness (measured as the mean number of MHC alleles per specimen) and microsatellite allelic richness in a given population. The Mantel test using the Spearman correlation and 10000 permutations between F_st_ distances for MHC and those for microsatellites was applied. The Spearman correlation was calculated between the mean individual values of q calculated for microsatellites and those calculated for MHC genes; the mean individual values of q represent the proportion of ancestry in cluster 1 or cluster 2 from the CLUMPP output using the Bayesian clustering approach. All statistical analyses were performed in Statistica 10.0 for Windows, StatSoft Inc, and R software (R version 2.12.2 (20011-02-25), The R Foundation for Statistical Computing using the package by Sarah Goslee and Dean Urban (2012) “Ecodist: Dissimilarity-based functions for ecological analysis”.

## Results

### Molecular profiles of Chondrostoma/Parachondrostoma populations

*Chondrostoma/Parachondrostoma* specimens from the localities investigated were genetically determined using mtDNA and 41 microsatellite loci. Using the combination of mtDNA and microsatellites, the allopatric status of a population for *C. nasus* at Allier and for *P. toxostoma* at Orbieu was confirmed. In the zones where *P. toxostoma* and *C. nasus* live in sympatry, our sampling revealed differences in the occurrence of both species between localities (Table 1). The number of hybrids within localities where these two species live in sympatry (i.e. Pont de Laragne, Pertuis, Manosque, Avignon, Saint Just and Labeaume) was variable, ranging from 3% to 20%.

**Table 1:** Populations of *P. toxostoma* (PT) and *C. nasus* (CN) studied plus sample sizes for hybrids (H) in sympatric zones.

Localities	Populations	Sample size	*DAB*-like alleles	Private alleles	*DAB1*-like alleles	*DAB3*-like alleles	*DAB*-like alleles per specimen
Orbieu	allopatric PT	11	6	2	1	5	1–4 (1.6, 0.92)
Allier	allopatric CN	12	9	1	4	5	1–4 (2.33,1.11)
Pertuis (Durance)	sympatric PT	18	17	0	7	10	1–5 (2.28, 1.19)
	sympatric CN	1	n. e.	0	n. e.	n. e.	1
	H	1	n. e.	1	n. e.	n. e.	2
Manosque (Durance)	sympatric PT	46	36	10	5	31	1–3 (2.02, 0.49)
	sympatric CN	8	10	0	3	7	2–4 (2.88, 0.60)
	H	10	12	2	3	9	2–3 (2.3, 0.46)
Avignon (Durance)	sympatric PT	-	-	-	-	-	-
	sympatric CN	34	25	1	6	19	1–5 (3.09,1.01)
	H	3	8	0	2	6	2–3 (3.33, 0.47)
Pont de Laragne (Buech)	sympatric PT	16	13	1	3	10	1–3 (2.06, 0.56)
	sympatric CN	17	22	3	4	18	1–6 (3, 1.28)
	H	5	12	0	4	8	1–5 (3, 1.26)
Labeaume (Ardeche)	sympatric PT	17	15	0	3	12	1–3 (1.88, 0.47)
	sympatric CN	-	-	-	-	-	-
	H	4	10	2	1	9	2–4 (3, 0.71)
Saint Just (Ardeche)	sympatric PT	11	19	5	4	15	1–3 (2, 0.60)
	sympatric CN	17	18	0	3	15	1–5 (3.18, 0.86)
	H	1	n. e.	0	n. e.	n. e.	3

The total number of *DAB*-like alleles, number of *DAB1*-like alleles and number of *DAB3*-like alleles in a population, and number of alleles per specimen (min - max, mean with standard deviation) are included. n. e. - not evaluated because of low sample size.

### MHC polymorphism in *P. toxostoma* and *C. nasus*: species level

*P. toxostoma* and *C. nasus* differ in their levels of MHC polymorphism measured by a spectrum of MHC alleles per species when all “pure” specimens identified using mtDNA and microsatellite markers were taken into account. A total of 55 different *DAB*-like alleles (13 *DAB1*-like and 42 *DAB3*-like) were identified in *P. toxostoma*. However, only 30 different *DAB*-like alleles (6 *DAB1*-like and 24 *DAB3*-like) were found in *C. nasus*. Concerning the alleles of the *DAB1* lineage, 8 *DAB1*-like alleles were specific to *P. toxostoma* (7 of them were found in at least two specimens and one was a singleton), and only one *DAB1*-like allele was specific to *C. nasus*. Concerning the alleles of the *DAB3* lineage, 34 alleles were specific to *P. toxostoma* (19 of them were found in at least two specimens and 15 of them were singletons), and 16 alleles were specific to *C. nasus* (12 of them were found in at least two specimens and 4 of them were singletons). Several alleles were shared by both species, i.e. 5 *DAB1*-like alleles and 8 *DAB3*-like alleles were found in the populations of both *P. toxostoma* and *C. nasus*. Some shared allelic variants appeared frequently in specimens (from 10% to 34% of specimens expressed a given sequence variant), whilst others were rarely found (from 0.8% to 7% of specimens expressed a given sequence variant). For all shared allelic variants except Pctn-DAB3*34 (this allelic variant was equally distributed between the two species), a high frequency was found in one species and a low frequency in the other (7 allelic variants were frequent in *C. nasus* and rare in *P. toxostoma*, whilst only 3 allelic variants were frequent in *P. toxostoma* and rare in *C. nasus*). Two shared alleles were also identified in the allopatric population of *P. toxostoma* and 5 shared alleles were also identified in the allopatric population of *C. nasus*. Despite the low proportions of hybrids in samples from the localities where both species live in sympatry, a high number of *DAB*-like alleles were introgressed into the hybrid genomes, i.e. 14 *P. toxostoma*-specific, 6 *C. nasus*-specific, and 7 shared *DAB*-like alleles were found in the hybrid specimens. The majority of shared allelic variants found in hybrids were frequent (see above), whilst species-specific allelic variants were found with variable frequency (0.9 to 12% of specimens expressed a given sequence variant). In addition, 6 other *DAB*-like alleles were recognized exclusively in the hybrid specimens.

**Figure 1:**
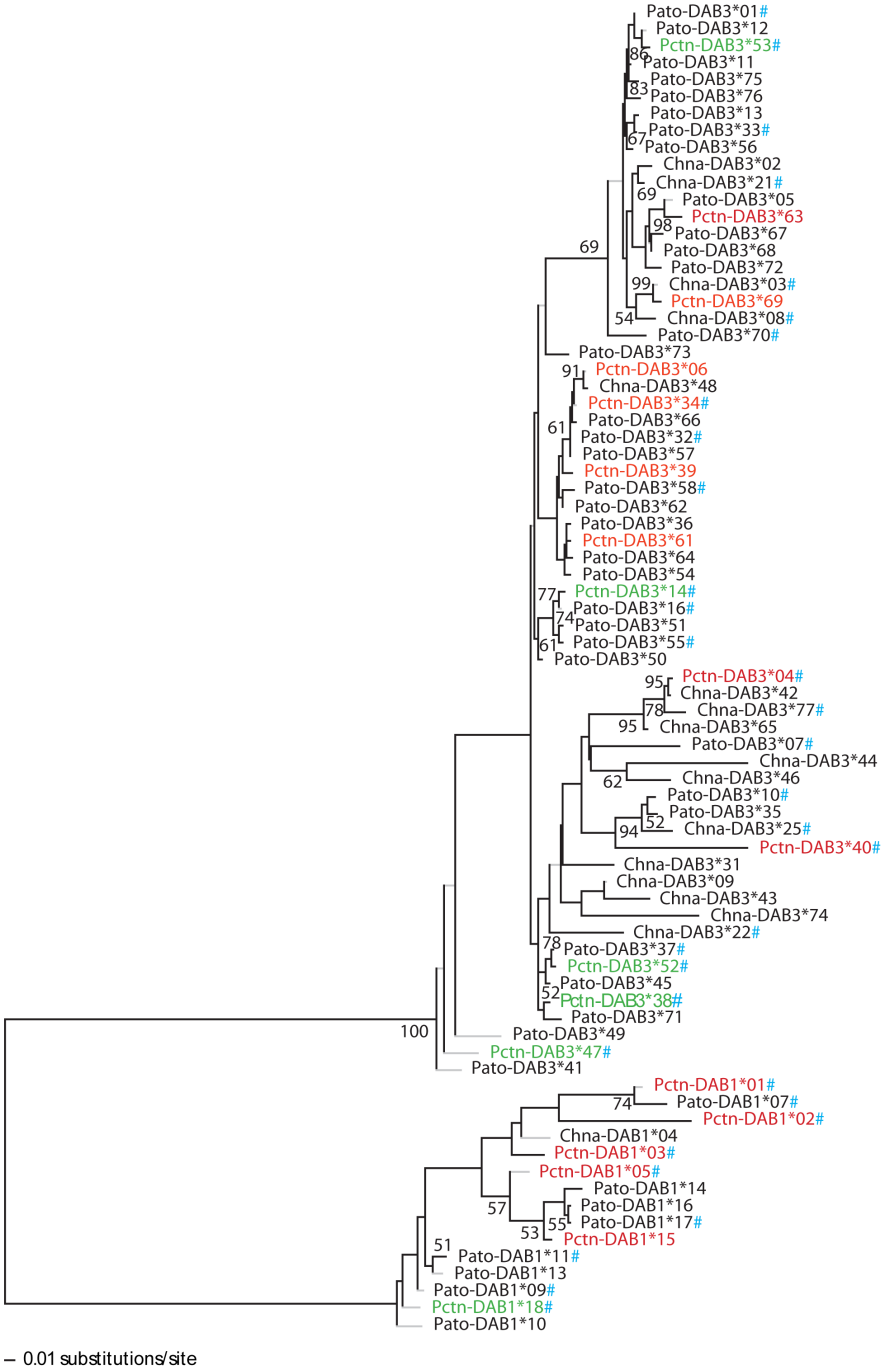
The neighbour-joining tree including all *DAB*-like alleles identified in *P. toxostoma* and *C. nasus*. The alleles found in pure specimens of both species, i.e. *P. toxostoma* (*Pato-DAB* alleles) and *C. nasus* (*Chna-DAB* alleles), are shown in black. The alleles shared between two species are shown in red, and those identified exclusively in hybrids are shown in green; both of them are termed as *Pctn-DAB* alleles. All alleles found in hybrids (i.e. species-specific, common or exclusively hybrid) are shown with #. Bootstrap values >50% are shown.

Phylogenetic reconstruction including all *DAB*-like alleles of *P. toxostoma*, *C. nasus* and their hybrids was performed to investigate the ancestry of MHC polymorphism in these fish. *DAB*-like alleles were clustered in two main lineages, i.e. *DAB1*-like and *DAB3*-like. Even though we confirmed the trans-species polymorphism between *P. toxostoma* and *C. nasus*, surprisingly, we found that *DAB3*-like alleles tended to cluster in a species-specific way, i.e. one large clade including a major proportion of *P. toxostoma*-specific alleles and one large clade including a major proportion of *C. nasus*-specific alleles were found (Figure 1). The alleles shared by both species were situated randomly within a phylogenetic tree. The position of alleles found exclusively in hybrids indicates that these alleles originated from *P. toxostoma*.

### MHC variability in *P. toxostoma*, *C. nasus* and hybrids: population and individual levels

**Figure 2:**
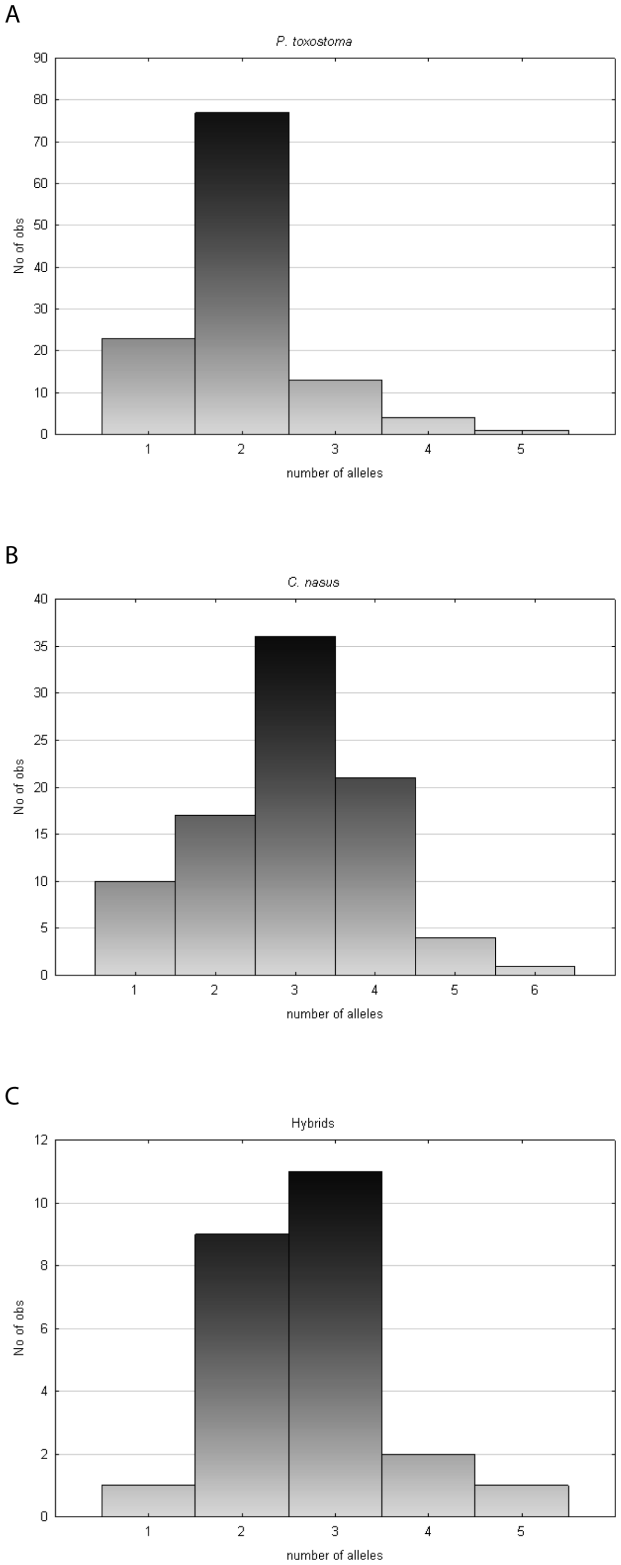
Distribution of *DAB*-like alleles in *P. toxostoma* (A), *C. nasus* (B), and hybrid (C) specimens.

In all populations of *C. nasus*, this species tended to reach a higher mean number of *DAB-*like alleles per specimen when compared with *P. toxostoma* populations (Table 1). *C. nasus* specimens expressed mainly three *DAB*-like alleles, whilst the majority of *P. toxostoma* specimens expressed two *DAB*-like alleles. The hybrids exhibited a pattern of *DAB*-like expression intermediate between *C. nasus* and *P. toxostoma*, i.e. the majority of them expressed two or three *DAB*-like alleles (Figure 2). The number of *DAB*-like alleles expressed in *C. nasus* was significantly higher than that expressed in *P. toxostoma* when including all specimens (from allopatric populations and from the sympatric zones), when including only the specimens from sympatric zones, or when analyzing separately the Durance and Ardeche sympatric zones (MW test, p<0.001).

**Figure 3:**
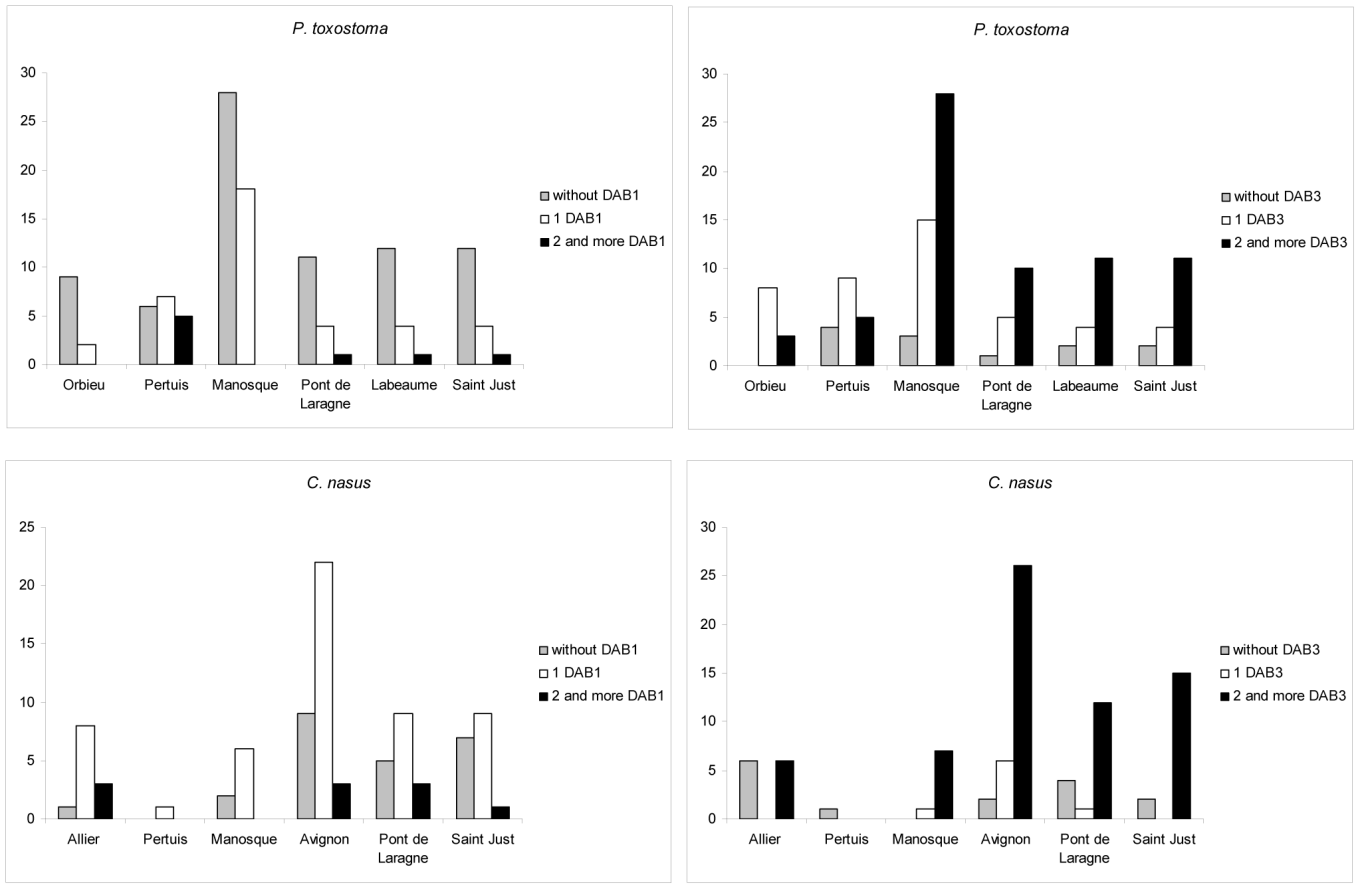
Frequency of the *DAB1* and *DAB3* expression patterns in *P. toxostoma* and *C. nasus* populations.

The numbers of different *DAB1*-like and *DAB3*-like alleles in the allopatric population of *C. nasus* was similar. However, the numbers of different *DAB3*-like alleles in the allopatric population of *P. toxostoma* and all sympatric populations of both species were higher than the numbers of *DAB1*-like alleles, and the same trend was observed for hybrids (Table 1). Using all populations or only those situated in sympatric areas, no effect of species on the number of different *DAB*-like alleles in the population was found (ANCOVA with sample size as covariate, p>0.05). Patterns of expression for the alleles of *DAB1* and *DAB3* lineages differed between *P. toxostoma* and *C. nasus* (Figure 3). Concerning the *DAB1* lineage, the proportion of *P. toxostoma* specimens from the allopatric population and the four populations sampled from sympatric areas that did not express the alleles of the *DAB1* lineage was the highest. Only in Pertuis (a locality within the Durance sympatric zone) were the proportions of *P. toxostoma* specimens expressing one *DAB1*-like allele, two *DAB1*-like alleles or no *DAB1*-like alleles balanced. On the other hand, the proportion of *C. nasus* specimens expressing one *DAB1*-like allele was the highest in all populations of this species. Concerning the *DAB3*-like lineage, the highest proportion of *P. toxostoma* specimens expressing at least two *DAB3*-like alleles was found in four populations sampled from sympatric areas, whilst the proportion of *P. toxostoma* specimens expressing a single *DAB3*-like allele was the highest in Orbieu (i.e. in the allopatric population) and Pertuis. The highest proportions of *C. nasus* specimens expressing at least two *DAB3*-like alleles were found in the samples from sympatric areas; the proportions of specimens without *DAB3*-like alleles or with at least two *DAB3*-like alleles in the sample from the allopatric population were low. In hybrids, 50% of specimens did not express the alleles of the *DAB1*-like lineage and 71% of them expressed at least two *DAB3*-like alleles.

### Recombination and selection maintaining MHC polymorphism

GENECONV detected four significant global inner fragments (sim p<0.05) in the *DAB*-like alignment of *P. toxostoma*. However, using Bonferroni-corrected KA p-values, no global inner fragment was significant (p>0.05). No significant global outer-sequence fragment was found. No significant inner or outer global fragments were detected in the *DAB*-like sequence alignment of *C. nasus*. Five additional inner pairwise fragments were significant (BC KA p-value p = 0.044).

The average rates of nonsynonymous substitutions were higher than those of synonymous ones for all sites and ABS sites for each species (Table 2). However, positive selection was not detected at all sites or at non-ABS sites. The *d*_N_/*d*_S_ ratio was significantly elevated only at ABS sites of *DAB*-like alleles in *P. toxostoma* and *C. nasus*, which provides evidence of positive selection acting on the PBS of MHC IIB of both species.

**Table 2:** The average rates of non-synonymous (d_N_) and synonymous (d_S_) substitutions (mean and standard errors obtained using 1000 bootstrap replicates), Z-test of positive selection with p-value included.

Species	Sites	d_N_	d_S_	Z	p
*P. toxostoma*	All sites	0.215 (0.028)	0.165 (0.036)	1.165	0.123
	ABS	0.500 (0.086)	0.188 (0.083)	3.443	<0.001
	non-ABS	0.138 (0.024)	0.158 (0.043)	−0.413	1.000
*C. nasus*	All sites	0.238 (0.031)	0.175 (0.033)	1.597	0.056
	ABS	0.591 (0.104)	0.246 (0.085)	3.319	0.001
	non-ABS	0.148 (0.023)	0.154 (0.038)	−0.143	1.000

**Figure 4:**
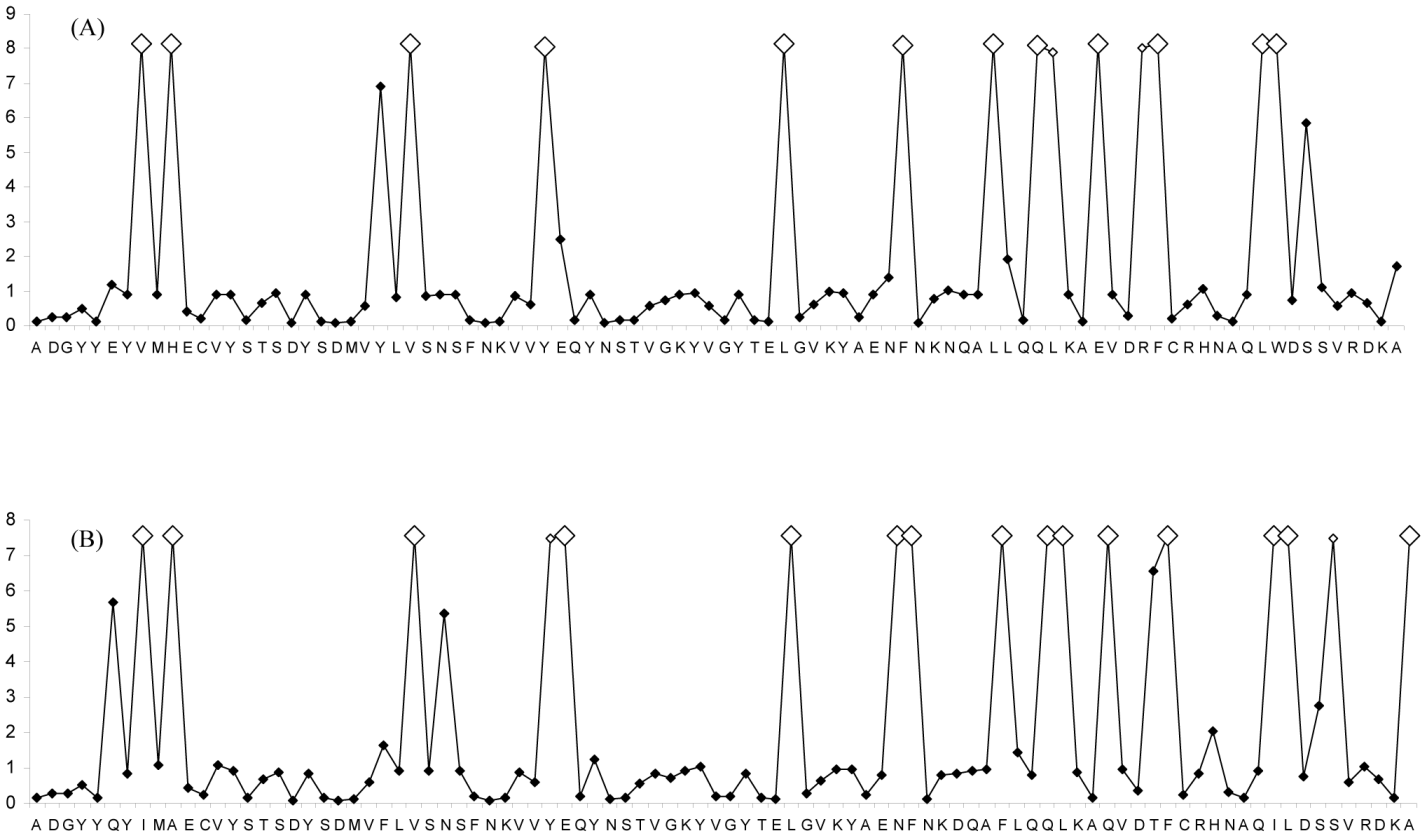
Approximate posterior means of ω under the M8 random sites model. Approximate posterior means of ω calculated as the weighted average of ω over the 11 site classes and weighted by the posterior probabilities under the M8 random sites model are shown for *DAB*-like sequence variants for *P. toxostoma* (A) and for *C. nasus* (B). Sites inferred to be under positive selection at the 99% level are indicated by large white squares and those at the 95% level are indicated by small white squares. The reference sequences in the graphic presentation are the following: Pato-DAB3*01 for *P. toxostoma* and Chna-DAB3*02 for *C. nasus*.

The LRT statistic comparing the two models indicates that the models that account for the sites under selection (i.e. M2a, M3 and M8) fit the data significantly better (*p*<0.001) than simpler models that do not allow for selection (i.e. M1a, M0 and M7), which indicates the action of positive selection at specific sites in *DAB* sequences. Log-likelihood values and parameter estimates under random-site models are included in Table 3. On the basis of BEB analysis, 17 PSS in *C. nasus* were identified using the M2a and M8 models, whilst only 12 sites using the M2a model and 14 sites using the M8 model were under positive selection in *P. toxostoma*. 14 out of 17 PSS in *C. nasus* and 12 out of 14 PSS in *P. toxostoma* corresponded to human antigen binding sites (ABS) identified using crystallography by Brown *et al.* (1993). The Bayes identification of sites under positive selection calculated using the M8 model is included in Figure 4. The same 13 PSS were identified in both species. In addition, codons 36, 58, 87 and 92 in *C. nasus* and codon 75 in *P. toxostoma* were identified to be under positive selection.

**Table 3:** Log-likelihood values and parameter estimates under random-site models for *P. toxostoma* and *C. nasus*.

Fish species	Model Code	log-likelihood	Estimates of parameters	Number of PSS
*P. toxostoma*	M0: one ratio (1)	−2751.964	ω = 2.198	
	M3: discrete (5)	−2500.823	p0 = 0.489, p1 = 0.437 , (p2 = 0.074), ω0 = 0.305, ω1 = 4.229, ω2 = 26.915	
	M1a: nearly neutral (1)	−2651.110	p0 = 0.509, (p1 = 0.491), (ω0 = 0.080), (ω1 = 1)	
	M2a: positive selection (3)	−2527.360	p0 = 0.357, p1 = 0.477, (p2 = 0.166), ω0 = 0.073, (ω1 = 1), ω2 = 7.929	9**, 3*
	M7: beta (2)	−2652.738	p = 0.244, q = 0.261	
	M8: beta and ω (4)	−2528.900	p0 = 0.831, (p1 = 0.169), p = 0.033, q = 0.019, ω = 8.265	12**,2*
*C. nasus*	M0: one ratio (1)	−2597.715	ω = 2.028	
	M3: discrete (5)	−2369.902	p0 = 0.526, p1 = 0.347, (p2 = 0.127), ω0 = 0.375, ω1 = 3.838, ω2 = 17.439	
	M1a: nearly neutral (1)	−2495.173	p0 = 0.536, (p1 = 0.464), (ω0 = 0.093), (ω1 = 1)	
	M2a: positive selection (3)	−2385.959	p0 = 0.33, p1 = 0.465, (p2 = 0.205), ω0 = 0.071, (ω1 = 1), ω2 = 7.524	13**, 4*
	M7: beta (2)	−2500.285	p = 0.234, q = 0.228	
	M8: beta and ω (4)	−2386.939	p0 = 0.792, (p1 = 0.208), p = 0.034, q = 0.020, ω = 7.761	15**, 2*

The number following the model code given in parentheses represents the number of free parameters for the ω ratios. Parameters in parentheses are presented for clarity only but are not free parameters; for example, under M8 *p*1 = 1-*p*0; PSS are the positive selected sites identified using the BEB method at.

* : P>95%;

** : P>99%;

ω is the selection parameter; p_n_ is the proportion of sites that fall into the ω_n_ site class; p and q are the shape parameters of the β function (for M7 and M8 models).

### Genetic structure of *P. toxostoma* and *C. nasus* populations

Of all 41 loci tested, a total of 16 microsatellite loci (LleA-029, LleA-071, BL1-98, BL2-114, Lsou08, Lsou34, Ppro132, CtoA-256, CtoE-249, CtoF-172, CypG24, IVO4, LCO1, N7K4, CA1 and Z21908) were discarded from the study due to the probable occurrence of null alleles as indicated by MICRO-CHECKER. All the remaining 25 loci that were subsequently used in genetic structure analyses were in HWE for each population.

**Figure 5:**
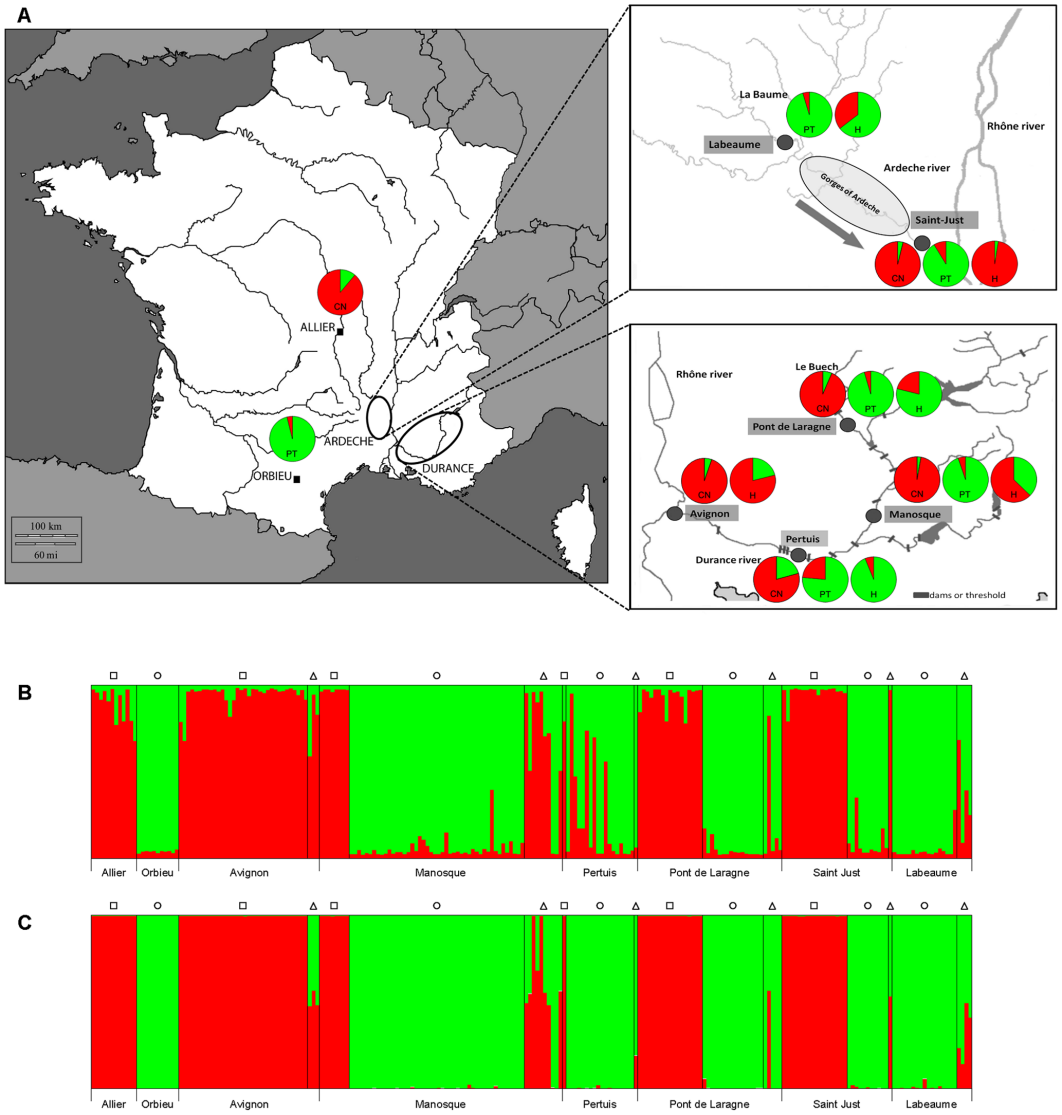
Genetic structure of *P. toxostoma* and *C. nasus* populations in MHC IIB genes and microsatellites. Genetic structure of *P. toxostoma* and *C. nasus* populations in MHC IIB genes (A, B) and microsatellites (C) inferred from Structure analysis for K = 2. The squares, circles and triangles correspond to *C. nasus*, *P. toxostoma* and hybrids identification based on the cyt *b* and microsatellites. Assignment of populations and hybrid samples to *P. toxostoma* and *C. nasus* clusters using MHC IIB genes is shown on the map (A).

Structure analysis using the Bayesian clustering approach revealed two genetic clusters when using microsatellites or MHC data without reflecting the pattern of geographical distribution or the origin of populations (i.e. the greater distances between allopatric and sympatric populations, the close proximity of sympatric populations, the different origins of the Durance hybrid zone and the Ardeche hybrid zone) (Figure 5). The analysis performed using microsatellites revealed the partial admixture between *P. toxostoma* and *C. nasus* in the areas where both species live in sympatry, confirming the hybridization process between these two species. Using MHC data, a more evident admixture between genetic clusters of *P. toxostoma* and *C. nasus* was observed when compared with microsatellite data. In the areas where both species live in sympatry, MHC data revealed bidirectional gene flow, as also indicated by microsatellites. The evident admixture of MHC alleles from *P. toxostoma* to *C. nasus* was observed even in the allopatric population of *C. nasus*. A higher admixture of MHC alleles from *C. nasus* to *P. toxostoma* than from *P. toxostoma* to *C. nasus* was observed in sympatric zones for specimens identified as hybrids based on the identification using microsatellites (especially evidenced in the locality Pertuis).

Pairwise F_st_ distances inferred from microsatellites revealed stronger genetic differentiation between *C. nasus* and *P. toxostoma* populations than that indicated by pairwise F_st_ distances inferred from MHC data. Based on microsatellites, both allopatric and sympatric populations of *C. nasus* were well differentiated from those of *P. toxostoma* (average pairwise F_st_ ranging from 0.415 to 0.524). Within species, higher F_st_ values were observed between the allopatric population and sympatric populations than between the pairs of sympatric populations (this trend was more evident in *P. toxostoma*) (see Supporting Information S3). Based on F_st_ calculated for binary encoded MHC data (Supporting Information S4), the allopatric population of *P. toxostoma* was much more different from its sympatric populations when compared to the allopatric and sympatric populations of *C. nasus*. The populations of *P. toxostoma* from sympatric zones were more similar than the allopatric and sympatric populations of this species. Such differences were less in evidence for *C. nasus.* The genetic differentiation based on MHC data calculated between sympatric populations of *P. toxostoma* and *C. nasus* was variable and independent of the origin of the populations (i.e. Durance versus Ardeche hybrid zone). Based on F_st_ calculated for haplotype MHC data (Supporting Information S4), the genetic differentiation between the *P. toxostoma* population from Pertuis and other *P. toxostoma* populations from Durance or Ardeche zones was higher than the genetic differentiation between other pairs of *P. toxostoma* populations from sympatric areas.

**Figure 6:**
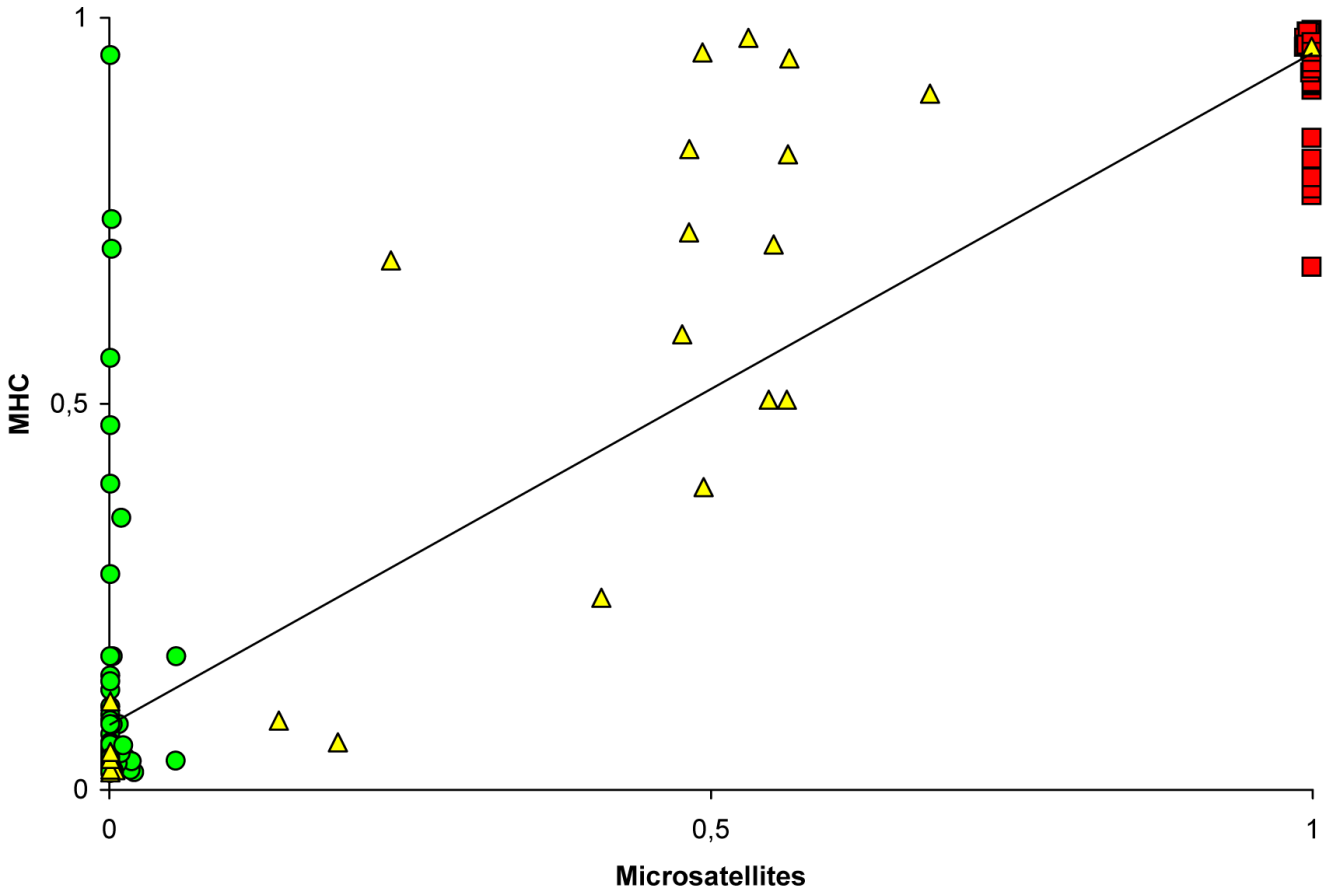
Probabilities assignation of specimens inferred from Structure using MHC and microsatellites. The red squares, green circles and yellow triangles correspond to *C. nasus*, *P. toxostoma* and hybrids identified using cyt *b* and microsatellites.

At population level, no effect of species on microsatellite diversity expressed as the number of different alleles or microsatellite allelic richness was found (ANCOVA with sample size as covariate, p>0.05). However, a significant effect of species on the mean number of MHC alleles per specimen in the population was found (ANCOVA with sample size as covariate, total F_2,8_ = 15.38, p = 0.002, sample size F = 1.00, p = 0.35, species F = 30.58, p<0.001) with a significantly higher MHC number in *C. nasus* populations when compared to *P. toxostoma* populations. At the population level, a positive correlation was found between microsatellite allelic richness (see Table 4) and mean number of MHC alleles per specimen in a given population (N = 11, R = 0.772, p = 0.005). A significant correlation between F_st_ distances calculated between pairs of populations for microsatellites and haplotype MHC data (N = 55, 0.507, p = 0.002) or between microsatellites and binary encoding MHC data (N = 55, R = 0.738, p<0.001) was found. The assigning of probabilities based on the individual values of q inferred from Structure using MHC and microsatellites is shown in Figure 6. At the individual level, a positive correlation was found between MHC and microsatellites based on the individual values of q inferred from structure analyses (N = 232, R = 0.831, p<0.001).

**Table 4:** Microsatellite diversity for *P. toxostoma* populations (PT), *C. nasus* populations (CN) and hybrid sample (H) including number of different alleles (N_a_), number of private alleles, allelic richness per sample with sample size >8 (i.e. per population or hybrid sample).

Localities	Populations	Sample size	N_a_	Private alleles	Allelic richness
Orbieu	allopatric PT	11	2.32	2	2.06
Allier	allopatric CN	12	3.80	5	3.19
Pertuis (Durance)	sympatric PT	18	5.24	4	3.13
	sympatric CN	1	1.60	0	n. e.
	H	1	1.52	6	n. e.
Manosque (Durance)	sympatric PT	46	7.20	13	3.14
	sympatric CN	8	4.40	3	3.50
	H	10	5.80	3	n. e.
Avignon (Durance)	sympatric PT	-	-	-	-
	sympatric CN	34	5.04	3	3.40
	H	3	3.40	3	n. e.
Pont de Laragne (Buech)	sympatric PT	16	4.84	2	3.10
	sympatric CN	17	4.28	2	3.28
	H	5	3.60	1	n. e.
Labeaume (Ardeche)	sympatric PT	17	5.04	4	3.18
	sympatric CN	-	-	-	-
	H	4	3.76	0	n. e.
Saint Just (Ardeche)	sympatric PT	11	4.28	3	3.14
	sympatric CN	17	4.72	3	3.47
	H	1	1.56	0	n. e.

n. e. – not evaluated.

## Discussion

The introduction of invasive species may be a factor influencing the genetic diversity of populations of native species, especially when considering endangered endemic species. This effect, however, though based on population genetics, has not been so extensively studied as habitat fragmentation or the isolation of endangered species. Therefore, we hypothesized that the introduction of *C. nasus* in southern France may represent a threat to native and endemic *P. toxostoma*, both found widely in sympatric areas and hybridizing, and focused on the variability in functional genes of high immunological importance such as MHC genes in these two phylogenetically-related cyprinid species, but species exhibiting distinct morphological and ecological traits.

### MHC diversity in *P. toxostoma* and *C. nasus*

Our analyses revealed different levels of MHC polymorphism in *P. toxostoma* and *C. nasus*. Native endemic *P. toxostoma* showed an extensive degree of MHC polymorphism measured by the number of different allelic variants per species when compared to *C. nasus*, probably resulting from the different origins and dispersion histories of both species. Concerning the populations of different endangered species, low genetic diversity of MHC especially due to a bottleneck effect has been shown (e.g. [3], [62]), suggesting that such species may be exposed to increased vulnerability to novel pathogens. However, *P. toxostoma*, a fish with protected status in southern France, expressed similar total numbers of MHC IIB allelic variants at the population level when compared to the populations of invasive *C. nasus*. This finding may suggest that similar evolutionary mechanisms operate to generate MHC diversity in both species (see below). However, at the individual level, a higher number of allelic variants were found for *C. nasus* specimens when compared to *P. toxostoma* specimens. Šimková *et al*. [32] demonstrated that *C. nasus* is a species more parasitized by monogenean species (i.e. this fish species is especially parasitized by the wider range of *Dactylogyrus* species that achieve a high intensity of infection) when compared to *P. toxostoma*. MHC genes are considered as possible candidates for co-adaptation genes evolving in interactions with parasite genotypes, especially those concerning host-specific parasites [63], [64]. The presence of a higher number of MHC allelic variants in *C. nasus* specimens compared to the number of MHC allelic variants in *P. toxostoma* specimens could result from co-evolutionary interactions between host and parasite genotypes and should explain the presence and high abundance of some monogenean parasite species in *C. nasus* and the low intensity of infection (or absence of some monogenean species) in *P. toxostoma*. However, further analyses are necessary to test such an evolutionary scenario.

Concerning the presence of *DAB*-like alleles of different allelic lineages, i.e. *DAB1*-like and *DAB3*-like previously recognized in different cyprinid fish species (e.g. [36], [41], [65], [66]), we confirmed the expression of alleles of *DAB1* and *DAB3* lineages in both *P. toxostoma* and *C. nasus*. In accordance with previous studies [66], [67], the specimens expressed the alleles of one lineage (i.e. *DAB1* or *DAB3*) or expressed the alleles of both. However, different patterns of expression were found when comparing native *P. toxostoma* and invasive *C. nasus*. Whilst the majority of *C. nasus* specimens expressed the alleles of both lineages, *P. toxostoma* specimens mainly expressed only the alleles of the *DAB3* lineage. Ottová *et al*. [67] showed that the expression of alleles of different *DAB* lineages is advantageous for fish males of common bream (*Abramis brama*), i.e. it is related to high fish condition and more intensive sexual coloration. Further immunoecological studies investigating whether the expression of the *DAB*-like alleles of the two different lineages represents some advantages potentially linked to body condition, immunity, survival or the dispersion of invasive *C. nasus* could be helpful in predicting the evolution of hybrid zones over time and, from a more general point of view, could also be helpful in clarifying the function of *DAB* genes in cyprinid fish species.

### MHC variability in hybrids

Our study indicated that the number of MHC variants found in *P. toxostoma* x *C. nasus* hybrid specimens is intermediate between the two parental species. Such a finding does not support the hypothesis of the super-optimal variability of MHC in hybrids, which was proposed as a mechanism selecting against interspecific hybrids by Eizaguirre *et al*. [68]. They hypothesized that if largely divergent sets of MHC alleles are combined from two mates of different species, an increased number of MHC variants in hybrid specimens may not only bind more foreign, but also a greater variety of self-derived peptides that are recognized by T cell clones. Thus, the T-cell repertoire available for parasite recognition is reduced. Therefore, hybrids should suffer from a higher parasite load, which was not, however, the case for *P. toxostoma* x *C. nasus* hybrids in our study. As shown by Šimková *et al*. [32], hybrids and *P. toxostoma* have similar intensities of ectoparasite infection, whilst *C. nasus* is more infected than *P. toxostoma* and the hybrids. This may suggest that the *P. toxostoma*-specific MHC alleles introgressed into the hybrid genomes may represent an advantage, i.e. such alleles probably generate low hybrid susceptibility to ectoparasites widely infecting invasive *C. nasus*.

Concerning the *DAB*-like alleles expressed in hybrids in our study, a higher number of alleles specific to *P. toxostoma* (which was the more diverse species concerning total MHC allelic diversity) were found in hybrids compared to the number of alleles specific to *C. nasus*; moreover, the phylogenetic reconstruction indicates that the alleles identified solely in hybrids were of *P. toxostoma* origin, suggesting that the hybrids are more introgressed by *P. toxostoma* than by *C. nasus*. This may indicate that the higher degree of MHC variability in hybrids originated from *P. toxostoma*. Nevertheless, many alleles shared between *P. toxostoma* and *C. nasus* were common in *C. nasus* and rare in *P. toxostoma*, and such alleles were also identified in hybrids, suggesting bidirectional MHC introgression. Bidirectional introgression, which is not common in hybridizing species, was previously demonstrated in these cyprinid fish using morphological traits, allozymes, and mtDNA [26], [27]. Following Bayesian cluster analysis using MHC data, *P. toxostoma* populations seem to be introgressed more often by *C. nasus* than *C. nasus* by *P. toxostoma* in sympatry. Surprisingly, a high level of introgression of MHC alleles from *C. nasus* was observed in the pure *P. toxostoma* population from Pertuis, i.e. in the specimens identified as *P. toxostoma* determined on the basis of mtDNA and microsatellites. In our study, all such specimens expressed three or four *DAB*-like alleles, which is an unusual situation for pure *P. toxostoma*. This may indicate that these specimens were the hybrids that we were not able to determine using 41 microsatellitte and mitochondrial markers. This result clearly demonstrates that different evolutionary forces influence the genomic structure of hybrid specimens. Indeed, a majority of microsatellite loci showed *P. toxostoma* alleles, and the *DAB* loci (MHC IIB genes) displayed *C. nasus* alleles. This result clearly shows how hybrid genotypes can serve as intermediaries for the transfer of adaptive genetic variation between parental populations. Such a pattern is well known in plants [69] but rarely demonstrated in vertebrates.

### MHC diversity: trans-species polymorphism, interspecific hybridization, or other factors

As already described, high MHC variability is a result of the action of several evolutionary mechanisms. Similarly to many previous studies performed on fish species (e.g. [36], [70]–[71]), we confirmed the trans-species evolution of MHC IIB genetic polymorphism in *P. toxostoma* and *C. nasus*, i.e. one *DAB3*-like allele (Pctn-DAB3*34) was shared between two species sampled in allopatric populations (however, only one allopatric population was sampled per species because of the protected status of *P. toxostoma* in southern France). In addition, the phylogenetic analyses revealed that some *C. nasus*-specific alleles were clustered within the groups of *P. toxostoma-*specific alleles, or that *P. toxostoma-*specific alleles were clustered within the groups of *C. nasus*-specific alleles. The other 12 alleles were found in both *P. toxostoma* and *C. nasus* in sympatric areas, which indicates that intergeneric hybridization plays an important role in sharing MHC alleles between *P. toxostoma* and *C. nasus* living in sympatry.

Nadachowska-Brzyska *et al*. [24], investigating two sister species of newts, showed that MHC variability in their parapatric ranges is increased due to interspecific hybridization. Nevertheless, Wegner and Eizaguirre [23] pointed out that it is difficult to distinguish between ancestral trans-species polymorphism and MHC introgression in species living and hybridizing in sympatry. In our study, MHC diversity seems to be shaped by both these processes. On the basis of Bayesian clustering using microsatellites, admixture of *P. toxostoma* and *C. nasus* genetic clusters was observed solely in the areas where both species live in sympatry, supporting the presence of intergeneric hybridization between the two cyprinid species. On the other hand, using MHC data, admixture was observed also in allopatric populations (in this case, the admixture of *P. toxostoma* into *C. nasus* was more evident), confirming that MHC diversity is shaped by trans-species polymorphism. However, a higher level of admixture was observed in areas where both species lived in sympatry, suggesting that intergeneric hybridization also contributes to MHC diversity in *P. toxostoma* and *C. nasus* populations. The role of intergeneric hybridization in increasing MHC variability in areas where both species live in sympatry is also supported by the fact that some hybrids carried alleles that were not identified in parental species. Thus, it could be suggested that intergeneric hybridization generates new MHC allelic variants by means of the crossover between non-sister chromatids during meiosis. New genomic combinations of *P. toxostoma* and *C. nasus* hybrids were also identified previously using nuclear data [27]. However, the presence of hybrid-specific alleles may also result from the limited sampling size in our study.

Nevertheless, in areas where both species live in sympatry, admixture was also observed when considering only the specimens of pure species (identified on the basis of mtDNA and 41 polymorphic microsatellite loci), which is also in line with the idea of the trans-species evolution of MHC polymorphism. However, a higher level of MHC polymorphism in sympatric populations when compared to MHC polymorphism in allopatric populations could also result from other factors such as habitat fragmentation and human activity linked with environmental pollution (e.g. Cohen [72]) or parasite-mediated selection (in our study, both species came into contact, which especially facilitated the transmission of ectoparasites). The introduction of a new parasite species following the introduction of its host fish could induce selection on the host immunity of native fish species in order to increase the number of MHC allelic variants within a population.

### Genetic differentiation of *P. toxostoma* and *C. nasus* populations

Using both the Bayesian clustering method and F_st_ distances, it was found that all populations of *P. toxostoma* were well differentiated from those of *C. nasus* on the basis of microsatellites. However, no genetic differentiation based on genetic clustering was evidenced between the allopatric population and all sympatric populations within each species. The allopatric population of *P. toxostoma* was well differentiated from all sympatric populations of this species based on F_st_ distances using microsatellites, whilst such genetic differentiation for *C. nasus* was little evident, which is in line with the recent history of the dispersion of invasive species (the Durance sympatric zone is about 100–150 years old, following Costedoat *et al*. [28], and hybridization in the Ardeche sympatric zone is of more recent origin, unpublished data). However, our results using binary encoded MHC data indicate stronger genetic differentiation between allopatric and sympatric populations of *P. toxostoma* than indicated by microsatellites, which may suggest that MHC in the allopatric population of native *P. toxostoma* investigated in our study is under different selective pressure than MHC in its sympatric populations. One of the possible explanations is the existence of different selective pressures from parasites. Šimková *et al*. [32] showed that the composition of parasite communities in the allopatric population of *P. toxostoma* from Orbieu is very different from that found in populations of *P. toxostoma* living in sympatry with *C. nasus*. Concerning invasive *C. nasus*, low genetic differentiation between the allopatric population and sympatric populations of this species was found: this differentiation was even comparable with the genetic differentiation between sympatric populations of *P. toxostoma* and *C. nasus*. Šimková *et al*. [32] revealed the highly similar composition of parasite communities between allopatric and sympatric populations of *C. nasus*, and the similar composition of parasite communities (based only on parasite presence data) in *P. toxostoma* and *C. nasus* populations living in sympatric areas. Nevertheless, to confirm the pattern of genetic differentiations between allopatric and sympatric populations of native *P. toxostoma* in further studies, it would be useful to include a wider range of allopatric populations for this species, which was not done in our study because of its threatened status.

### Positive versus neutral selection acting on MHC genes

Using a combination of specific functional loci (such as MHC genes) and genome-wide random markers (such as microsatellite loci) is considered to be a good way to assess the threat posed by reduced genetic diversity in species [5]. Such an approach combining microsatellites and MHC analyses was performed for several endangered species and revealed reduced microsatellite and MHC diversity and/or a positive correlation between MHC variation and the variation at neutral loci across populations, indicating that genetic drift significantly affects MHC variability on a short timescale and that even the genetic drift in some small bottlenecked and fragmented populations may overshadow the role of balancing selection [6]–[7], [73]. However, several studies identifying the effect of genetic drift on MHC diversity also demonstrated the significant role of balancing selection in shaping MHC polymorphism on a long timescale (see the review study on MHC diversity by Radwan *et al*. [20]). In our study, we showed a positive correlation between microsatellite allelic richness and mean number of MHC alleles per individual in a population, suggesting the effect of neutral selection on MHC variability in *C. nasus* and *P. toxostoma* populations. In addition, a positive correlation between measurements of microsatellite variability and MHC diversity was found at the individual level. Nevertheless, our analyses also indicated strong positive selection acting on PBS in both species. The maximum likelihood codon-based analyses, which were performed to detect whether MHC IIB in each species was under selection and to identify the PSS, revealed a similar ω-ratio in the *DAB*-like genes of both species, indicating a similar strength of positive selection acting on MHC in native and invasive species. The majority of sites identified under positive selection were the same in both species and corresponded well to ABS identified for humans by Brown *et al*. [50], even if the total number of PSS was slightly higher in invasive *C. nasus* when compared with endemic *P. toxostoma*. This invasive species was also found to be more parasitized by *Chondrostoma*-specific parasites than *P. toxostoma* and recombinant genotypes of *C. nasus* and *P. toxostoma* hybrids [32].

## Supporting Information

Supporting Information S1 The alignment of *DAB* genes' amplicons and reference database sequences (“A” for *DAB1*-like genes and “B” for *DAB3*-like genes) containing complete sequence of exon 2 and flanking partial sequences of exons 1 and 3. The region of complete exon 2 in both genes (276 bp long) is represented by representative alleles of *DAB1*-like and *DAB3*-like genes (Pctn-DAB1*01 and Pato-DAB3*01; respectively). The identical positions of both genes and reference sequences are shaded. The figure was constructed using alignment in MEGA 5 [49] and edited in BioEdit v. 7.0.9.0 [74] and Adobe Photoshop CS software.
(DOC)

Supporting Information S2 **Summary tables (1, 2 and 3) of the validation procedure for 454 pyrosequencing data sets.** Table 1 – Quality Control Data, Table 2 – Roche NGS report data and data from SESAME Assistant Analysis, Table 3 – Genetic variation in DAB sequences. The present data were obtained from study report of each run (Tables 1 and 2). Quality of the pools (Table 1) were checked by size range analysis using a DNA 1000 Assay on the 2100 Bioanalyzer (Agilent Technologies) and quantified by fluorescent measurement using the Quant-it™ Picogreen® DNA assay (Invitrogen) and Nanodrop 8000 (ThermoScientific) and performed by the provider (Beckman Coulter Genomics). *The modal read length is the most frequent read length seen with a moving window of 7 bases (Table 2). Data of genetic variation (Table 3) were obtained by SESAME analysis.
(DOC)

Supporting Information S3 **The values of pairwise F_st_ distances (average with 95% confidence intervals are shown) for microsatellites.** The names of the localities and population assignment i.e. CN – *C. nasus*, PT – *P. toxostoma* are included.
(DOC)

Supporting Information S4 **The values of pairwise F_st_ distances (average with 95% confidence intervals are shown) for MHC using binary encoded data (below the diagonal) and haplotype frequencies (above the diagonal).** The names of the localities and population assignment i.e. CN – *C. nasus*, PT – *P. toxostoma* are included.
(DOC)
