# Supplementary figures and images for: Genomic Porosity between Invasive Chondrostoma nasus and Endangered Endemic Parachondrostoma toxostoma (Cyprinidae): The Evolution of MHC IIB Genes
Source: PLoS One. 2013 Jun 18;8(6):e65883. doi: 10.1371/journal.pone.0065883 (PMC3688810; doi:10.1371/journal.pone.0065883)

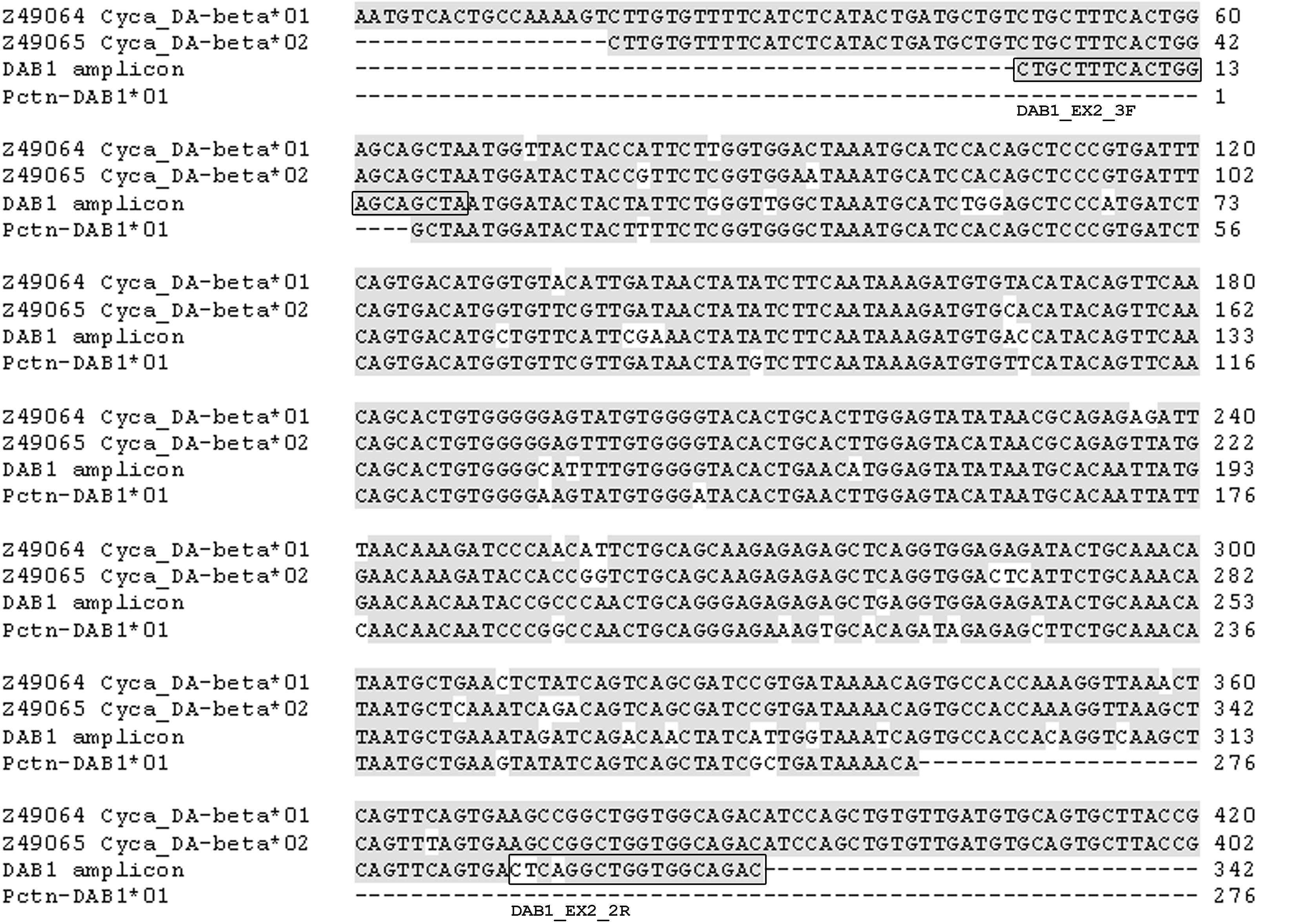


**(A)**


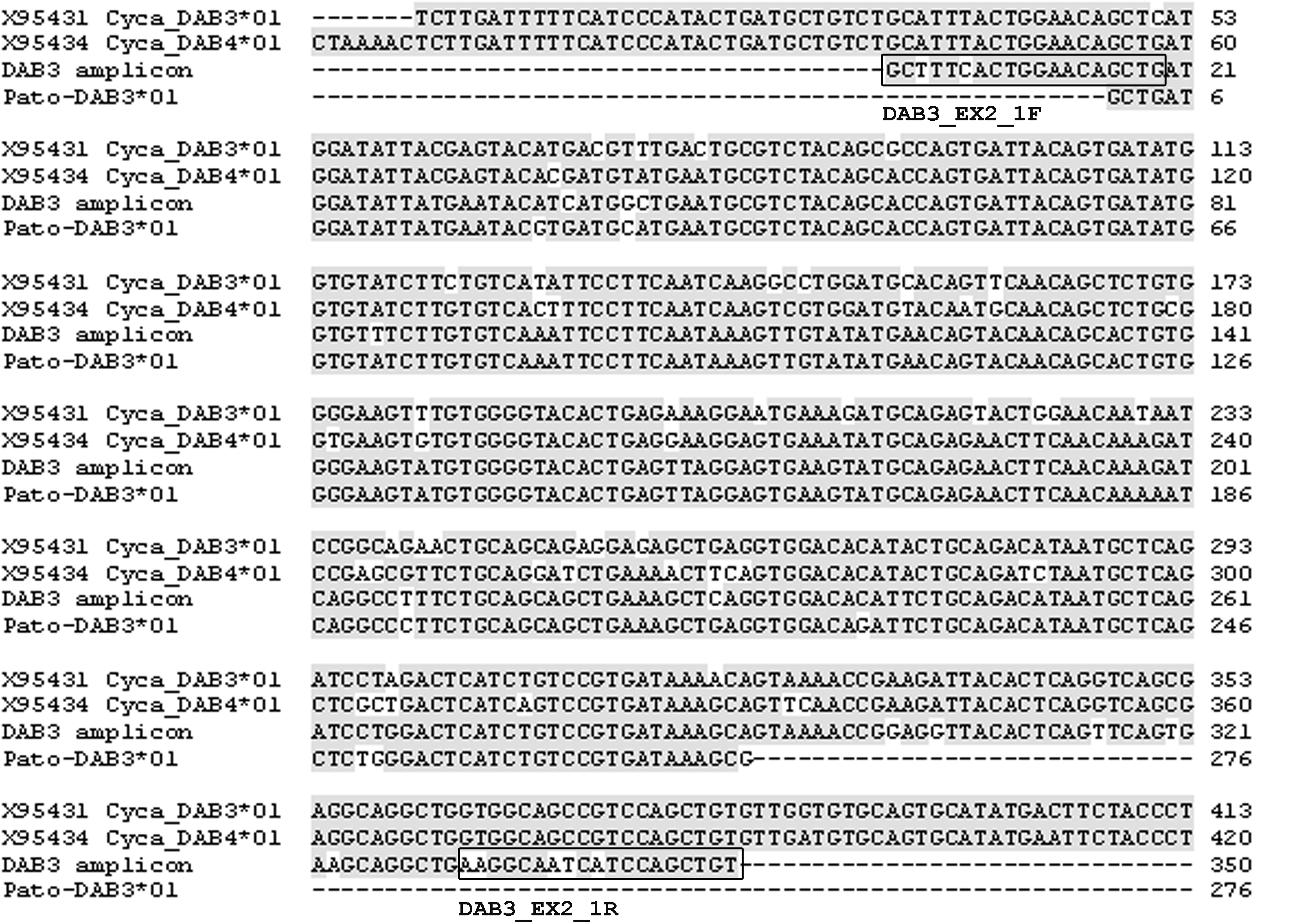


**(B)**

Supplement: Supporting Information S1 — The alignment of DAB genes' amplicons and reference database sequences (“A” for DAB1-like genes and “B” for DAB3-like genes) containing complete sequence of exon 2 and flanking partial sequences of exons 1 and 3. The region of complete exon 2 in both genes (276 bp long) is represented by representative alleles of DAB1-like and DAB3-like genes (Pctn-DAB1*01 and Pato-DAB3*01; respectively). The identical positions of both genes and reference sequences are shaded. The figure was constructed using alignment in MEGA 5 [49] and edited in BioEdit v. 7.0.9.0 [74] and Adobe Photoshop CS software. (DOC) [file pone.0065883.s001.doc]
