# Supplementary material for: Genomic Porosity between Invasive Chondrostoma nasus and Endangered Endemic Parachondrostoma toxostoma (Cyprinidae): The Evolution of MHC IIB Genes
Source: PLoS One. 2013 Jun 18;8(6):e65883. doi: 10.1371/journal.pone.0065883 (PMC3688810; doi:10.1371/journal.pone.0065883)
Supplement: Supporting Information S2 — Summary tables (1, 2 and 3) of the validation procedure for 454 pyrosequencing data sets. Table 1 – Quality Control Data, Table 2 – Roche NGS report data and data from SESAME Assistant Analysis, Table 3 – Genetic variation in DAB sequences. The present data were obtained from study report of each run (Tables 1 and 2). Quality of the pools (Table 1) were checked by size range analysis using a DNA 1000 Assay on the 2100 Bioanalyzer (Agilent Technologies) and quantified by fluorescent measurement using the Quant-it™ Picogreen® DNA assay (Invitrogen) and Nanodrop 8000 (ThermoScientific) and performed by the provider (Beckman Coulter Genomics). *The modal read length is the most frequent read length seen with a moving window of 7 bases (Table 2). Data of genetic variation (Table 3) were obtained by SESAME analysis. (DOC) [file pone.0065883.s002.doc]

**Supporting information S2.**

| Table 1 | DNA pool concentration |  | Nano:Pico ratio | Ammount of DNA (ug) | 260/280 | 260/230 |
| --- | --- | --- | --- | --- | --- | --- |
|  | Nano (ng/ul) | Pico (ng/ul) |  |  |  |  |
| Analysis run 1 | 23.5 | 10.82 | 2.2 | 4.33 | 1.78 | 0.77 |
| Analysis run 2 | 22.19 | 5.5 | 4.0 | 4.4 | 1.55 | 0.71 |
| Analysis run 3 | 16.8 | 3.5 | 4.8 | 1.96 | 1.62 | 0.59 |

| Table 2 | Samples in DNA pool | Roche Mb | Roche Modal* (bp) | Roche Median (bp) | Raw sequences (Roche Reads) | Markers-assigned sequences (eValue=1E-10) | Samples-assigned sequences | Samples with sequences | Variants |
| --- | --- | --- | --- | --- | --- | --- | --- | --- | --- |
|  |  |  |  |  |  |  |  |  |  |
| Analysis run 1 | 190 | 63.3 | 356 | 344 | 214469 | 197966 | 87581 | 188 | 24714 |
| Analysis run 2 | 152 | 40.6 | 364 | 363 | 116241 | 112476 | 100079 | 150 | 24217 |
| Analysis run 3 | 128 | 102.6 | 364 | 363 | 288506 | 282533 | 254719 | 128 | 45814 |

| Table 3 |  | Number of samples in DNA pool (control samples) | Mean number of reads per sample (min/max values) | Mean number of variants per sample (min/max values) |
| --- | --- | --- | --- | --- |
| Analysis run 1 | DAB1 | 94 (33) | 507.6 (0/1204) | 143.5 (0/289) |
|  | DAB3 | 96 (32) | 415.2 (0/1032) | 117.0 (0/314) |
| Analysis run 2 | DAB1 | 56 (7) | 664.3 (0/1416) | 168.9 (0/382) |
|  | DAB3 | 96 (8) | 655.0 (0/2764) | 153.7 (0/468) |
| Analysis run 3 | DAB1 | 32 (4) | 2764.4 (41/6800) | 496.1 (24/1130) |
|  | DAB3 | 96 (4) | 1731.7 (57/10099) | 311.9 (17/1512) |
