# Supplementary material for: Genomic Porosity between Invasive Chondrostoma nasus and Endangered Endemic Parachondrostoma toxostoma (Cyprinidae): The Evolution of MHC IIB Genes
Source: PLoS One. 2013 Jun 18;8(6):e65883. doi: 10.1371/journal.pone.0065883 (PMC3688810; doi:10.1371/journal.pone.0065883)
Supplement: Supporting Information S3 — The values of pairwise Fst distances (average with 95% confidence intervals are shown) for microsatellites. The names of the localities and population assignment i.e. CN – C. nasus, PT – P. toxostoma are included. (DOC) [file pone.0065883.s003.doc]

**Supporting information S3.**

|  | Allier CN | Orbieu PT | Avignon CN | Manosque CN | Manosque PT | Pertuis PT | Pont de Laragne CN | Pont de Laragne PT | Saint Just CN | Saint Just PT | Labeaume PT |
| --- | --- | --- | --- | --- | --- | --- | --- | --- | --- | --- | --- |
| Allier CN | - |  |  |  |  |  |  |  |  |  |  |
| Orbieu PT | 0.520  (0.427 - 0.609) | - |  |  |  |  |  |  |  |  |  |
| Avignon CN | 0.038  (0.023 - 0.059) | 0.476  (0.390 - 0.564) | - |  |  |  |  |  |  |  |  |
| Manosque CN | 0.053  (0.027 - 0.079) | 0.524  (0.428 - 0.623) | 0.003  (-0.009 - 0.015) | - |  |  |  |  |  |  |  |
| Manosque PT | 0.475  (0.378 - 0.591) | 0.172  (0.115 - 0.231) | 0.452  (0.35 - 0.557) | 0.479  (0.368 - 0.593) | - |  |  |  |  |  |  |
| Pertuis PT | 0.456  (0.353 - 0.561) | 0.167  (0.104 - 0.233) | 0.42754  (0.326 - 0.528) | 0.457  (0.345 - 0.572) | 0.001  (-0.003 - 0.006) | - |  |  |  |  |  |
| Pont de Laragne CN | 0.042  (0.024 - 0.064) | 0.505  (0.418 - 0.597) | 0.006  (-0.002 - 0.014) | 0.002  (-0.008 - 0.013) | 0.473  (0.37197 - 0.580) | 0.448  (0.345 - 0.553) | - |  |  |  |  |
| Pont de Laragne PT | 0.460  (0.363 - 0.560) | 0.161  (0.109 - 0.216) | 0.431  (0.337 - 0.525) | 0.460  (0.354 - 0.567) | 0.006  (0.001 - 0.012) | 0.002  (-0.008 - 0.014) | 0.452  (0.357 - 0.551) | - |  |  |  |
| Saint Just CN | 0.045  (0.029 - 0.068) | 0.487  (0.399 - 0.579) | 0.002  (-0.007 - 0.011) | 0.012  (-0.009 - 0.037) | 0.457  (0.354 - 0.566) | 0.432  (0.32812 - 0.539) | 0.021  (0.009 - 0.032) | 0.434  (0.336 - 0.535) | - |  |  |
| Saint Just PT | 0.444  (0.347 - 0.543) | 0.168  (0.116 - 0.225) | 0.415  (0.321 - 0.512) | 0.440  (0.336 - 0.548) | 0.004  (-0.003 - 0.012) | -0.004  (-0.010 - 0.001) | 0.435  (0.339 - 0.533) | 0.004  (-0.006 - 0.017) | 0.415  (0.318 - 0.516) | - |  |
| Labeaume PT | 0.449  (0.349 - 0.555) | 0.165  (0.114 - 0.223) | 0.422  (0.323 - 0.522) | 0.448  (0.338 - 0.562 | 0.008  (0.002 - 0.014) | -0.001  (-0.006 - 0.005) | 0.442  (0.342 - 0.547) | 0.014  (0.002 - 0.028) | 0.423  (0.319 - 0.529) | -0.001  (-0.010 - 0.011) | - |
