# Supplementary material for: Genomic Porosity between Invasive Chondrostoma nasus and Endangered Endemic Parachondrostoma toxostoma (Cyprinidae): The Evolution of MHC IIB Genes
Source: PLoS One. 2013 Jun 18;8(6):e65883. doi: 10.1371/journal.pone.0065883 (PMC3688810; doi:10.1371/journal.pone.0065883)
Supplement: Supporting Information S4 — The values of pairwise Fst distances (average with 95% confidence intervals are shown) for MHC using binary encoded data (below the diagonal) and haplotype frequencies (above the diagonal). The names of the localities and population assignment i.e. CN – C. nasus, PT – P. toxostoma are included. (DOC) [file pone.0065883.s004.doc]

**Supporting information S4.**

|  | Allier CN | Orbieu PT | Avignon CN | Manosque CN | Manosque PT | Pertuis PT | Pont de Laragne CN | Pont de Laragne PT | Saint Just CN | Saint Just PT | Labeaume PT |
| --- | --- | --- | --- | --- | --- | --- | --- | --- | --- | --- | --- |
| Allier CN | - | 0.225  (0.202 - 0.247) | 0.005  (0.001 - 0.009) | 0.042  (0.034 - 0.049) | 0.145  (0.128 - 0.162) | 0.012  (0.004 - 0.021) | 0.024  (0.020 - 0.029) | 0.133  (0.118 - 0.148) | 0.083  (0.073 - 0.094) | 0.117  (0.102 - 0.132) | 0.126  (0.110 - 0.143) |
| Orbieu PT | 0.335  (0.117 - 0.541) | - | 0.152  (0.131 - 0.173) | 0.160  (0.129 - 0.191) | 0.112  (0.085 - 0.137) | 0.227  (0.209 - 0.246) | 0.133  (0.108 - 0.159) | 0.119  (0.091 - 0.147) | 0.143  (0.112 - 0.175) | 0.093  (0.067 - 0.118) | 0.110  (0.081 - 0.139) |
| Avignon CN | 0.043  (-0.006 - 0.099) | 0.332  (0.062 - 0.516) | - | 0.034  (0.026 - 0.041) | 0.082  (0.069 - 0.095) | 0.033  (0.025 - 0.043) | 0.009  (0.006 - 0.012) | 0.071  (0.061 - 0.082) | 0.048  (0.042 - 0.054) | 0.054  (0.044 - 0.065) | 0.067  (0.053 - 0.079) |
| Manosque CN | 0.087  (-0.027 - 0.211) | 0.485  (0.113 - 0.688) | -0.003  (-0.037 - 0.037) | - | 0.091  (0.067 - 0.115) | 0.115  (0.098 - 0.134) | -0.017  (-0.020 - -0.013) | 0.084  (0.062 - 0.107) | -0.018  (-0.020 - -0.016) | 0.062  (0.041 - 0.083) | 0.071  (0.049 - 0.093) |
| Manosque PT | 0.165  (0.094 - 0.232) | 0.240  (0.020 - 0.463) | 0.238  (0.090 - 0.382) | 0.288  (0.071 - 0.492) | - | 0.126  (0.112 - 0.138) | 0.059  (0.045 - 0.074) | -0.015  (-0.016 - -0.014) | 0.087  (0.065 - 0.112) | -0.010  (-0.014 - -0.002) | -0.014  (-0.016 - -0.012) |
| Pertuis PT | 0.142  (0.068 - 0.205) | 0.285  (0.066 - 0.499) | 0.215  (0.074 - 0.344) | 0.272  (0.064 - 0.471) | 0.084  (0.017 - 0.154) | - | 0.076  (0.065 - 0.090) | 0.119  (0.106 - 0.131) | 0.149  (0.131 - 0.168) | 0.112  (0.099 - 0.124) | 0.115  (0.103 - 0.127) |
| Pont de Laragne CN | -0.025  (-0.041 - -0.004) | 0.259  (0.060 - 0.449) | 0.023  (-0.011 - 0.071) | 0.015  (-0.062 - 0.108) | 0.159  (0.088 - 0.224) | 0.112  (0.049 - 0.166) | - | 0.050  (0.038 - 0.063) | 0.002  (-0.001 - 0.005) | 0.032  (0.021 - 0.046) | 0.042  (0.029 - 0.056) |
| Pont de Laragne PT | 0.199  (0.145 - 0.244) | 0.306  (0.076 - 0.523) | 0.253  (0.106 - 0.390) | 0.323  (0.114 - 0.535) | -0.001  (-0.021 - 0.026) | 0.116  (0.051 - 0.172) | 0.164  (0.096 - 0.219) | - | 0.077  (0.056 - 0.098) | -0.017  (-0.022 - -0.012) | -0.022  (-0.024 - -0.020) |
| Saint Just CN | 0.110  (0.033 - 0.182) | 0.388  (0.100 - 0.564) | 0.005  (-0.013 - 0.024) | -0.015  (-0.049 - 0.023) | 0.269  (0.100 - 0.430) | 0.252  (0.102 - 0.398) | 0.046  (-0.003 - 0.096) | 0.284  (0.123 - 0.444) | - | 0.055  (0.036 - 0.075) | 0.070  (0.048 - 0.092) |
| Sain tJust PT | 0.118  (0.058 - 0.169) | 0.238  (-0.010 - 0.483) | 0.189  (0.035 - 0.339) | 0.256  (0.027 - 0.488) | 0.029  (-0.012 - 0.072) | 0.066  (0.027 - 0.098) | 0.094  (0.031 - 0.149) | 0.035  (-0.013 - 0.090) | 0.223  (0.054 - 0.396) | - | -0.022  (-0.026 - -0.017) |
| Labeaume PT | 0.179  (0.130 - 0.218) | 0.277  (0.035 - 0.510) | 0.227  (0.101 - 0.353) | 0.292  (0.093 - 0.488) | 0.031  (-0.005 - 0.065) | 0.079  (0.032 - 0.118) | 0.142  (0.091 - 0.183) | 0.026  (-0.011 - 0.071) | 0.257  (0.113 - 0.403) | 0.013  (-0.021 - 0.052) | - |
